# Supplementary material for: Single-molecule imaging and microfluidic platform reveal molecular mechanisms of leukemic cell rolling
Source: Commun Biol. 2021 Jul 14;4:868. doi: 10.1038/s42003-021-02398-2 (PMC8280113; doi:10.1038/s42003-021-02398-2)
Supplement: Supplementary file 2 — Supplementary Information [file 42003_2021_2398_MOESM2_ESM.pdf]

Supplementary Information for

# Single-Molecule Imaging and Microfluidic Platform Reveal Molecular Mechanisms of Leukemic Cell Rolling

*Bader Al Alwan[1], Karmen AbuZineh[1], Shuho Nozue[1], Aigerim Rakhmatulina[1], Mansour Aldehaiman[1], Asma S. Al-Amoodi[1], Maged F. Serag[1], Fajr A. Aleisa[1], Jasmeen S. Merzaban[1,2,*], and Satoshi Habuchi[1,2,*]*

[1] King Abdullah University of Science and Technology (KAUST), Biological and Environmental Science and Engineering Division, Thuwal 23955-6900, Saudi Arabia

[2] These authors contributed equally to the work

[*] Correspondence should be addressed to S.H. or J.S.M.

## Supplementary Figures

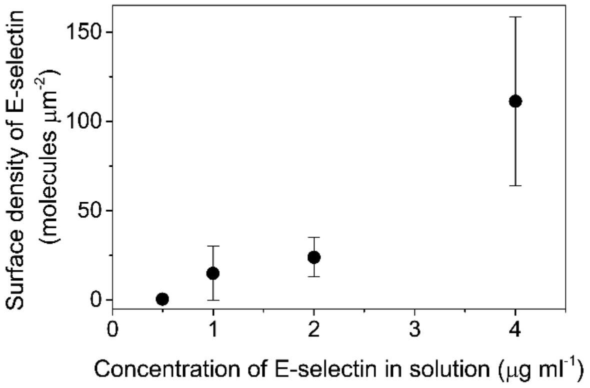


**Supplementary Figure 1. Surface density of rh E-selectin molecules.** The surface densities of the rh E-selectin molecules were determined after incubating the microfluidic chambers with HBSS buffer containing varied concentrations of the recombinant E-selectin at 4 °C overnight. The error bars show the standard deviations determined by at least four separate experiments.

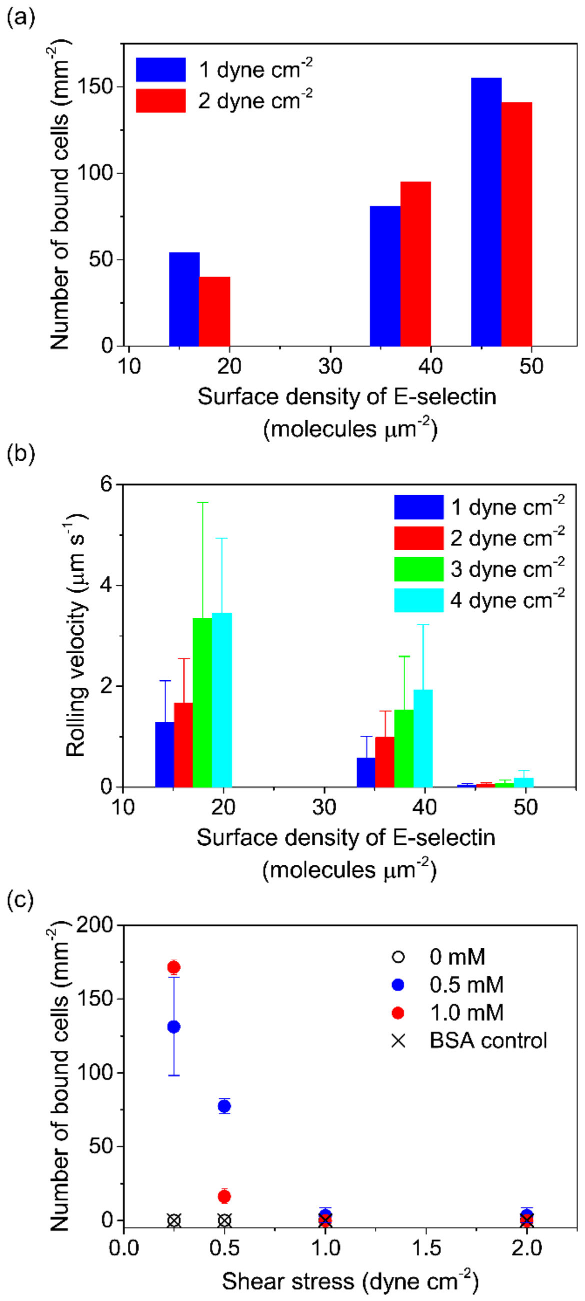


**Supplementary Figure 2. Characterization of the rolling behavior of KG1a cells on E-selectin. (a)** Number of bound cells to the E-selectin surface at varied surface densities of the deposited rh E-selectin. The cells were injected into the microfluidic chambers at the shear stresses of either 1 or 2 dyne cm$^{-2}$ (0.1 or 0.2 Pa). **(b)** Rolling velocity of the cells on the E-selectin surface at different surface densities of the deposited rh E-selectin. The cells were injected into the

microfluidic chambers at the shear stresses of either 1, 2, 3, or 4 dyne $cm^{-2}$ (0.1, 0.2, 0.3, or 0.4 Pa). The error bars show the standard deviations determined by at least six separate experiments. **(c)** Number of bound cells to the E-selectin surface at different applied shear stresses. The cells were injected into the microfluidic chambers at the calcium ion concentrations of either 0, 0.5, or 1.0 mM. BSA control (i.e. no E-selectin on the surface) is included as a negative control. The error bars show the standard deviations determined by at least three separate experiments.

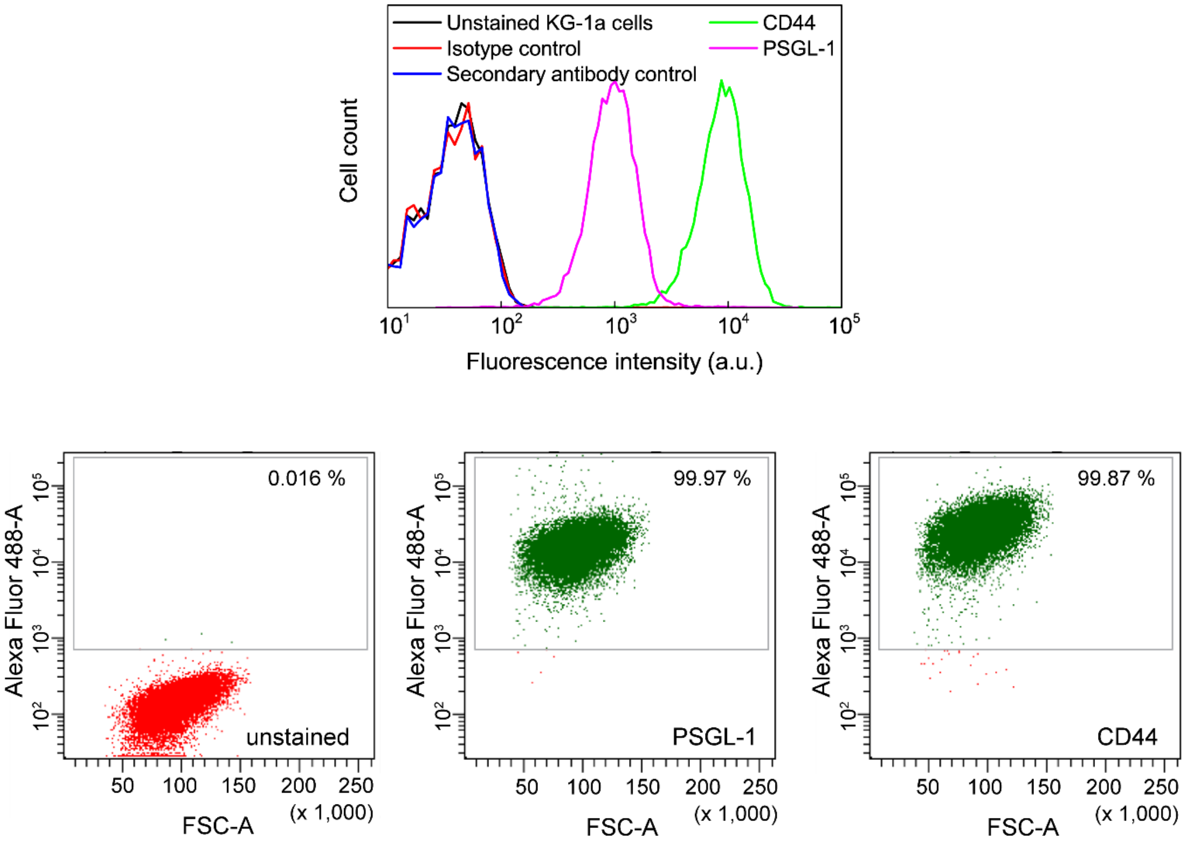


**Supplementary Figure 3. Flow cytometric analysis of the binding specificity of CD44 and PSGL-1 antibodies.** CD44 (green) and PSGL-1 (magenta) on KG1a cells were immunostained by antibodies against CD44 and PSGL-1 (clone 515 for CD44 and clone KPL-1 for PSGL-1) and Alexa-Fluor-488-conjugated secondary antibody against the isotype of the CD44 and PSGL-1 antibodies (goat anti-mouse IgG). Unstained KG1a cells (black), isotype control labelled cells (red), and secondary alone-labelled cells (blue) were included as controls. This is a representative experiment of n=2 independent experiments.

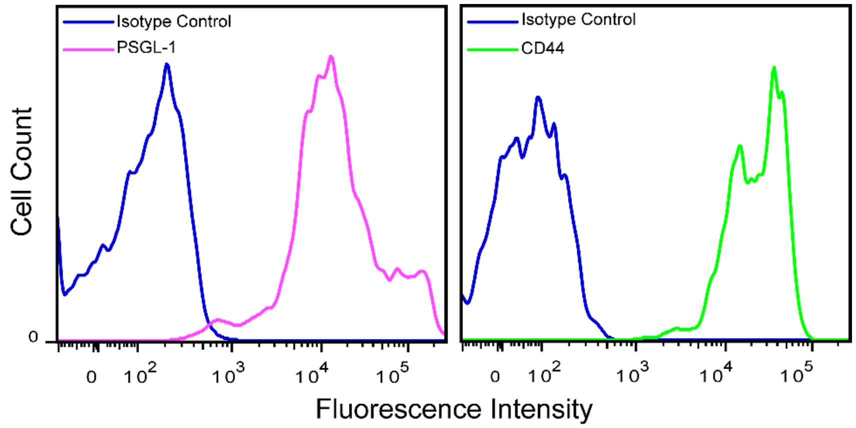


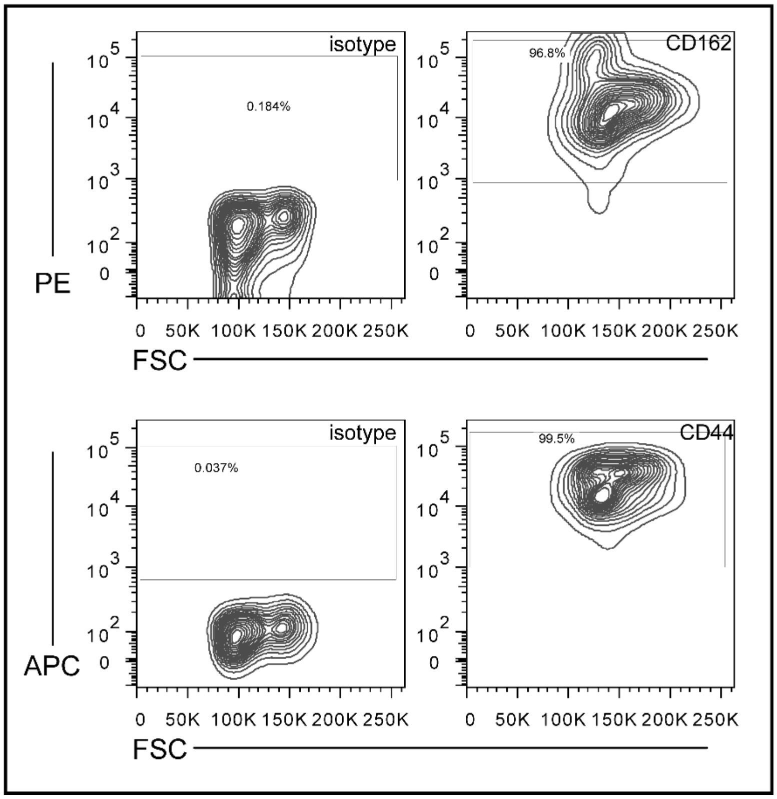


**Supplementary Figure 4. Assessment of E-selectin ligand expression on human CD34$^{pos}$-HSPCs.** Primary human CD34$^{pos}$-HSPCs were stained for CD44 (green) and PSGL-1 (magenta) using antibodies specific to these antigens (anti-human CD44, clone 2C5 and anti-human PSGL-1, clone KPL-1) and analyzed by flow cytometry (FACS Canto and FlowJo). This is a representative experiment of n=2 independent experiments.

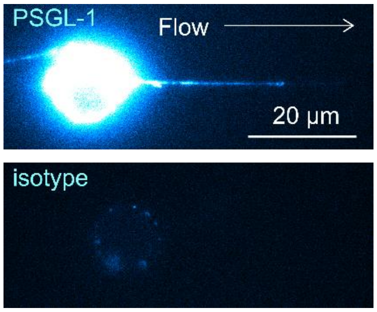


**Supplementary Figure 5. Binding specificity of the antibody characterized by fluorescence imaging.** (top) Fluorescence image of PSGL-1 molecules on a KG1a cell that were immunostained by Alexa-Fluor-555-conjugated antibody (anti-PSGL-1 antibody, clone KPL-1). The labeled cells were injected into the rh E-selectin-deposited microfluidic chambers. The cells were injected into the chambers at a shear stress of 2 dyne $cm^{-2}$ (0.2 Pa). (bottom) Fluorescence image of an isotype control labelled KG1a cell that was injected into the fluidic chambers at conditions identical to those for the PSGL-1 immunostained cells.

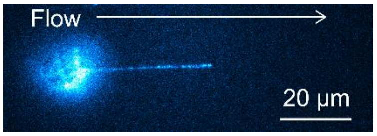


**Supplementary Figure 6.** Immunofluorescence image of PSGL-1 on KG1a cell captured using the Fab fragment of the anti-PSGL-1 antibody. The PSGL-1 molecules on the KG1a cells were immunostained by the Alexa-Fluor-555-conjugated Fab fragment of the anti-PSGL-1 antibody (KPL-1 clone). The fluorescence image was recorded during the KG1a cells rolling over E-selectin at a shear stress of 2 dyne cm-2 (0.2 Pa).

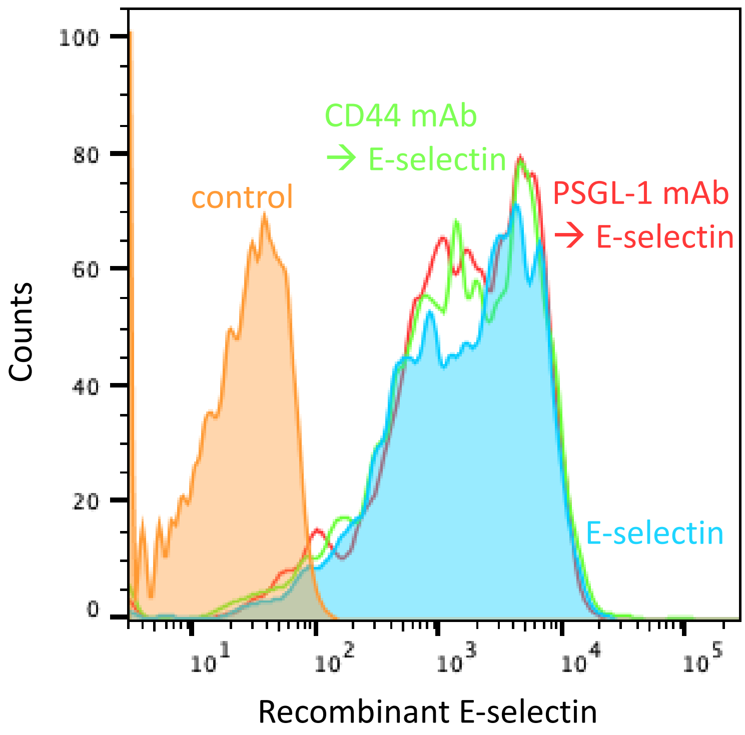


**Supplementary Figure 7. Flow cytometric analysis of the binding of E-selectin to the KG1a cells in the presence of an antibody to CD44 or PSGL-1.** KG1a cells were either incubated with antibodies to CD44 (green), PSGL-1 (red), or without (cyan). Following a washing step, all populations were then incubated with recombinant E-selectin. Control cells represent secondary antibody to E-selectin.

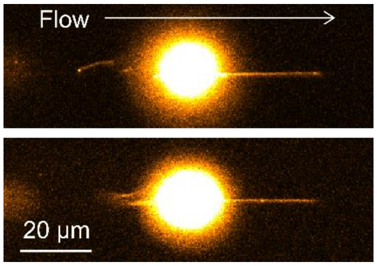


**Supplementary Figure 8. Formation of tethers and slings on KG1a cells while rolling over E-selectin.** Examples of the fluorescence images of the cell membrane (stained by Vybrant DiO dye) captured during cell rolling over the surface-deposited rh E-selectin molecules. The cells were injected into the chambers at a shear stress of 2 dyne $cm^{-2}$ (0.2 Pa).

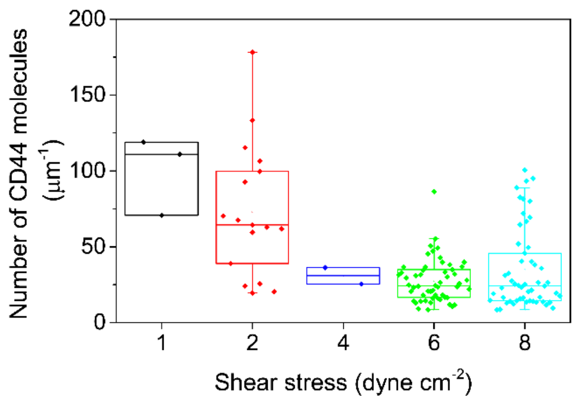


**Supplementary Figure 9.** Number of CD44 molecules per unit length of slings. The CD44 molecules on the KG1a cells were immunostained by the Alexa-Fluor-647-conjugated anti-CD44 antibody (515 clone). The fluorescence images were recorded during KG1a cell rolling over E-selectin at shear stresses of 1, 2, 4, 6, and 8 dynes cm$^{-2}$ (0.1, 0.2, 0.4, 0.6, and 0.8 Pa).

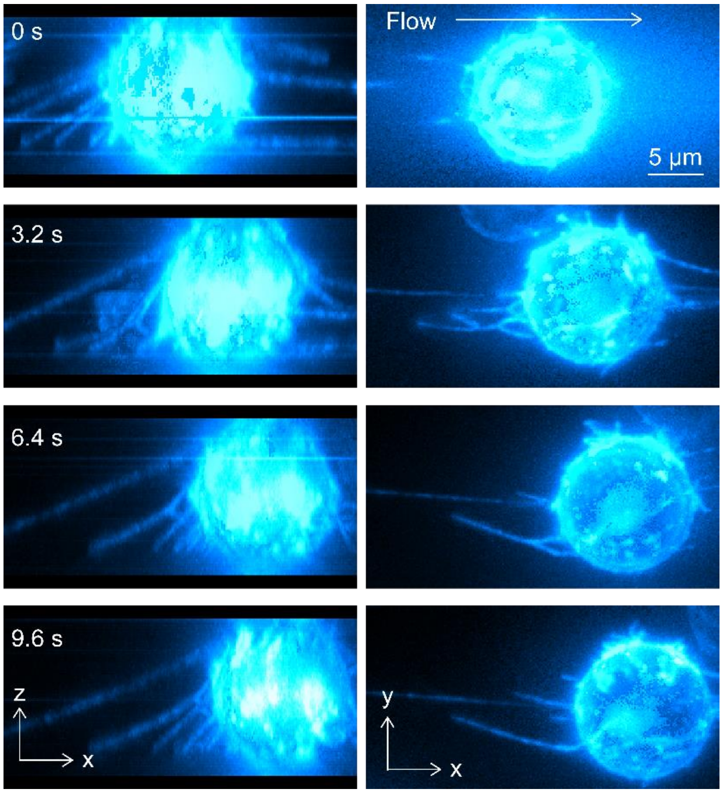


**Supplementary Figure 10. 3D views of the tethers and slings formed on a KG1a cell rolling over E-selectin by CD44.** Side view (left) and top view (right) of the 3D reconstructed time-lapse fluorescence images of CD44 (immunostained by Alexa-Fluor-647-conjugated anti-CD44 antibody, clone 515) captured during cell rolling over the surface-deposited rh E-selectin molecules. The 3D images were reconstructed by recording fluorescence images of the cell at 53 different Z-axis positions with 0.5 μm step size. The cells were injected into the chambers at a shear stress of 2 dyne $cm^{-2}$ (0.2 Pa).

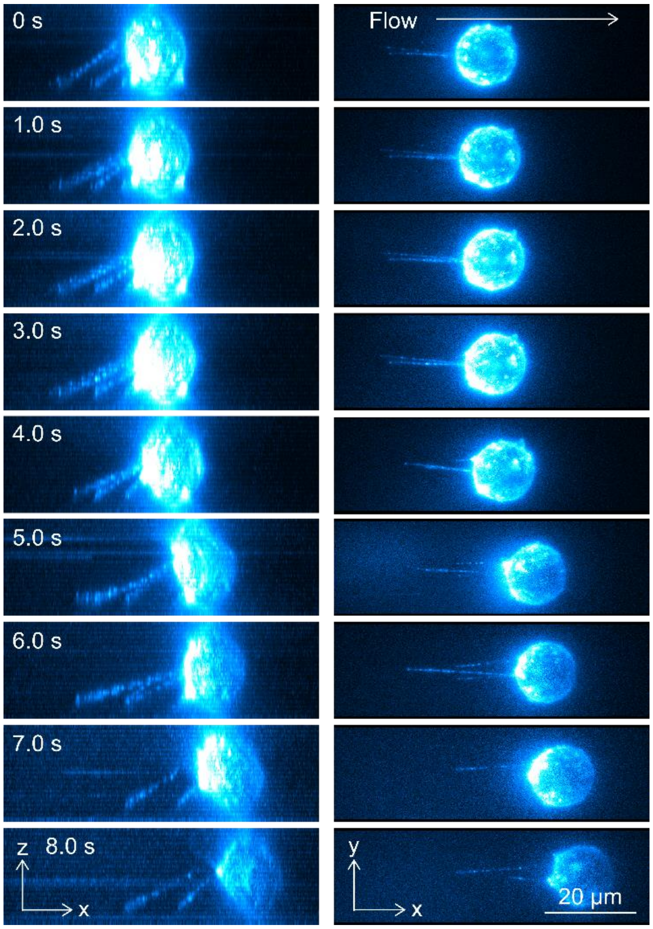


**Supplementary Figure 11. 3D views of the tethers formed on a KG1a cell rolling over E-selectin by PSGL-1.** Side view (left) and top view (right) of the 3D reconstructed time-lapse fluorescence images of PSGL-1 (immunostained by Alexa-Fluor-555-conjugated anti-PSGL-1 antibody, clone KPL-1) captured during cell rolling over surface-deposited rh E-selectin molecules. The 3D images were reconstructed by recording fluorescence images of the cell at 33 different Z-axis positions with 1.0 μm step size. The cells were injected into the chambers at a shear stress of 2 dyne $cm^{-2}$ (0.2 Pa).

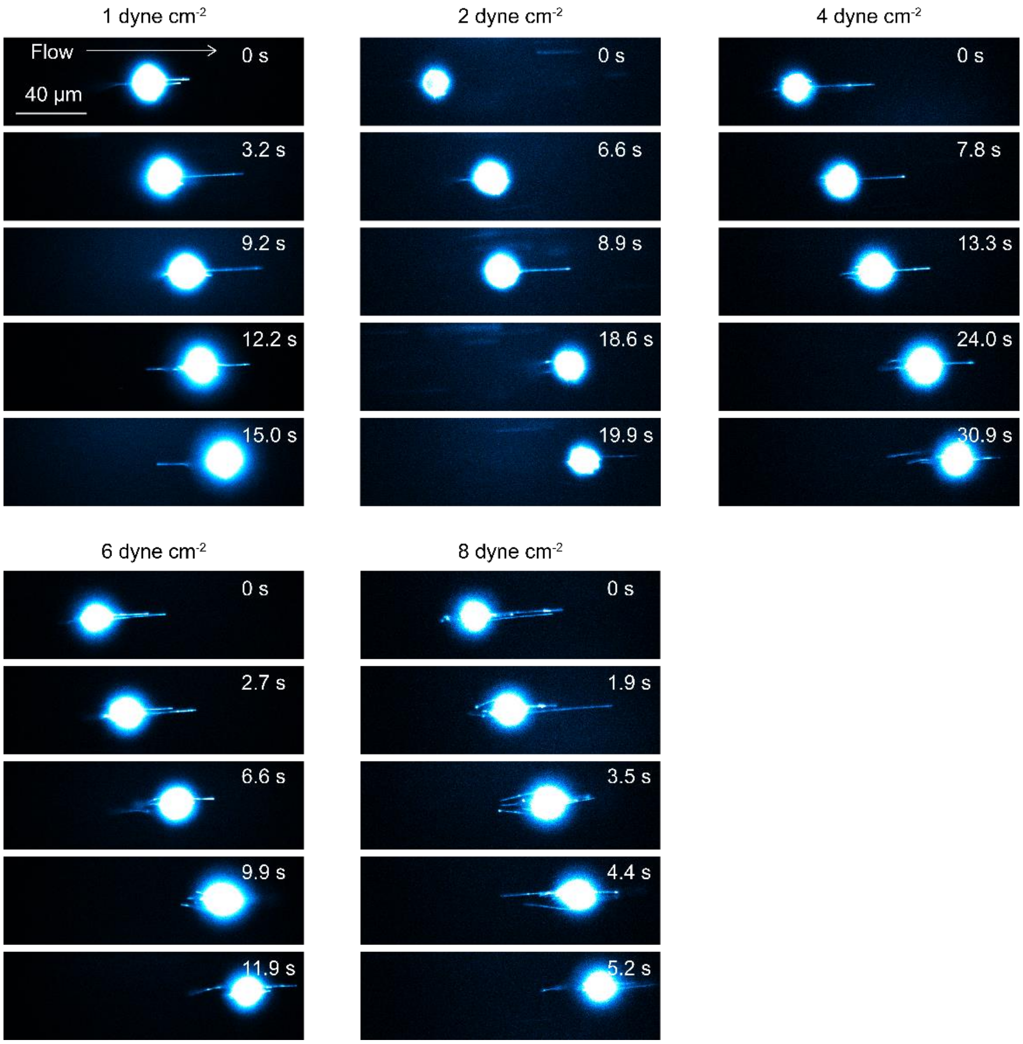


**Supplementary Figure 12. Shear force-dependent formation of the tethers and slings on KG1a cells rolling over E-selectin.** Time-lapse fluorescence images of CD44 (immunostained by Alexa-Fluor-647-conjugated anti-CD44 antibody clone 515) captured during cell rolling over the surface-deposited rh E-selectin molecules. The cells were injected into the chambers at a shear stress of 1, 2, 4, or 8 dyne cm$^{-2}$ (0.1, 0.2, 0.4, or 0.8 Pa).

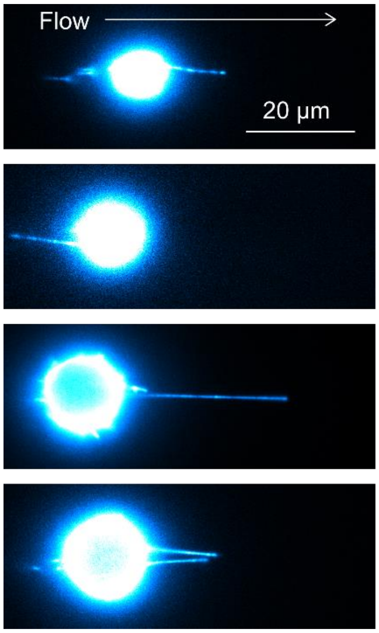


**Supplementary Figure 13. Formation of the tethers and slings on primary human CD34$^{pos}$-HSPCs rolling over E-selectin.** Examples of the fluorescence images of CD44 (immunostained by Alexa-Fluor-647-conjugated anti-CD44 antibody, clone 515) captured during human CD34$^{pos}$-HSPC rolling over the surface-deposited rh E-selectin molecules. The cells were injected into the chambers at a shear stress of 2 dyne cm$^{-2}$ (0.2 Pa).

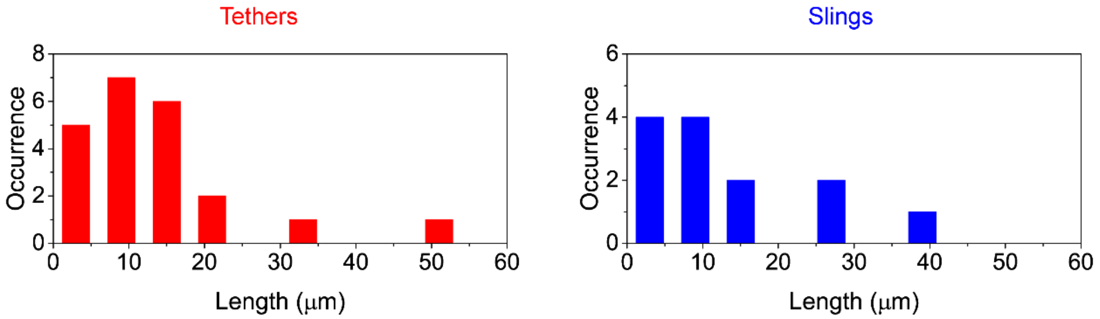


**Supplementary Figure 14. Length of the tethers and slings formed on primary human CD34$^{pos}$-HSPCs rolling over E-selectin.** Frequency histograms of the length of tethers (red bars) and slings (blue bars) formed during the primary human CD34$^{pos}$-HSPCs rolling over the surface-deposited rh E-selectin. The cells were injected into the chambers at a shear stress of 2 dyne cm$^{-2}$ (0.2 Pa) as indicated.

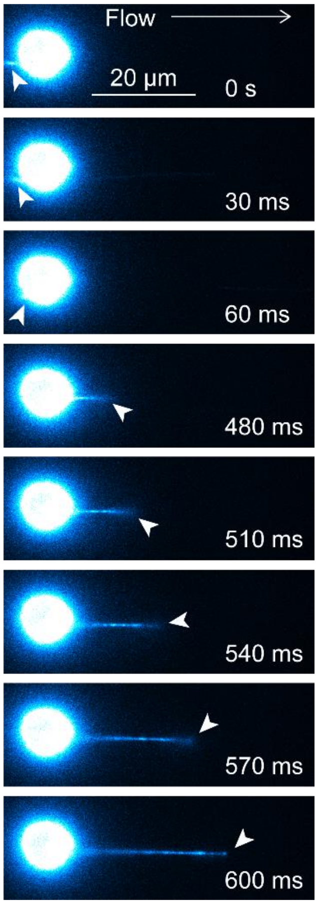


**Supplementary Figure 15. Conversion of a tether into sling observed for a primary human CD34pos-HSPC rolling over E-selectin.** Time-lapse fluorescence images of CD44 (immunostained by Alexa-Fluor-647-conjugated anti-CD44 antibody, clone 515) captured during cell rolling over the surface-deposited rh E-selectin molecules. The arrow heads show the tether that is converted into sling upon the detachment of the tethering point from the E-selectin surface. The cells were injected into the chambers at a shear stress of 2 dyne $cm^{-2}$ (Pa).

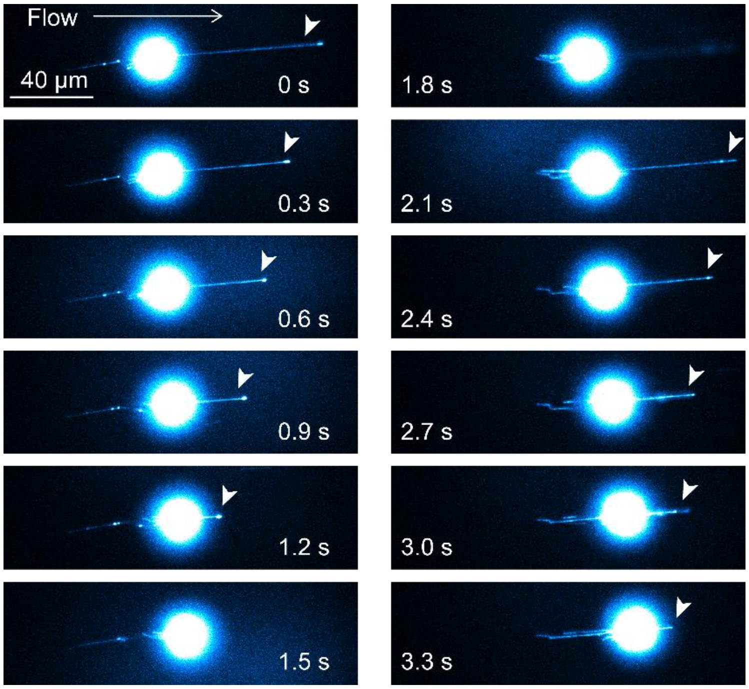


**Supplementary Figure 16. Retraction of the slings.** Time-lapse fluorescence images of CD44 (immunostained by Alexa-Fluor-647-conjugated anti-CD44 antibody, clone 515) captured during cell rolling over the surface-deposited rh E-selectin molecules. The arrow heads show the slings with this retraction behavior. The cells were injected into the chambers at a shear stress of 8 dyne $cm^{-2}$ (0.8 Pa).

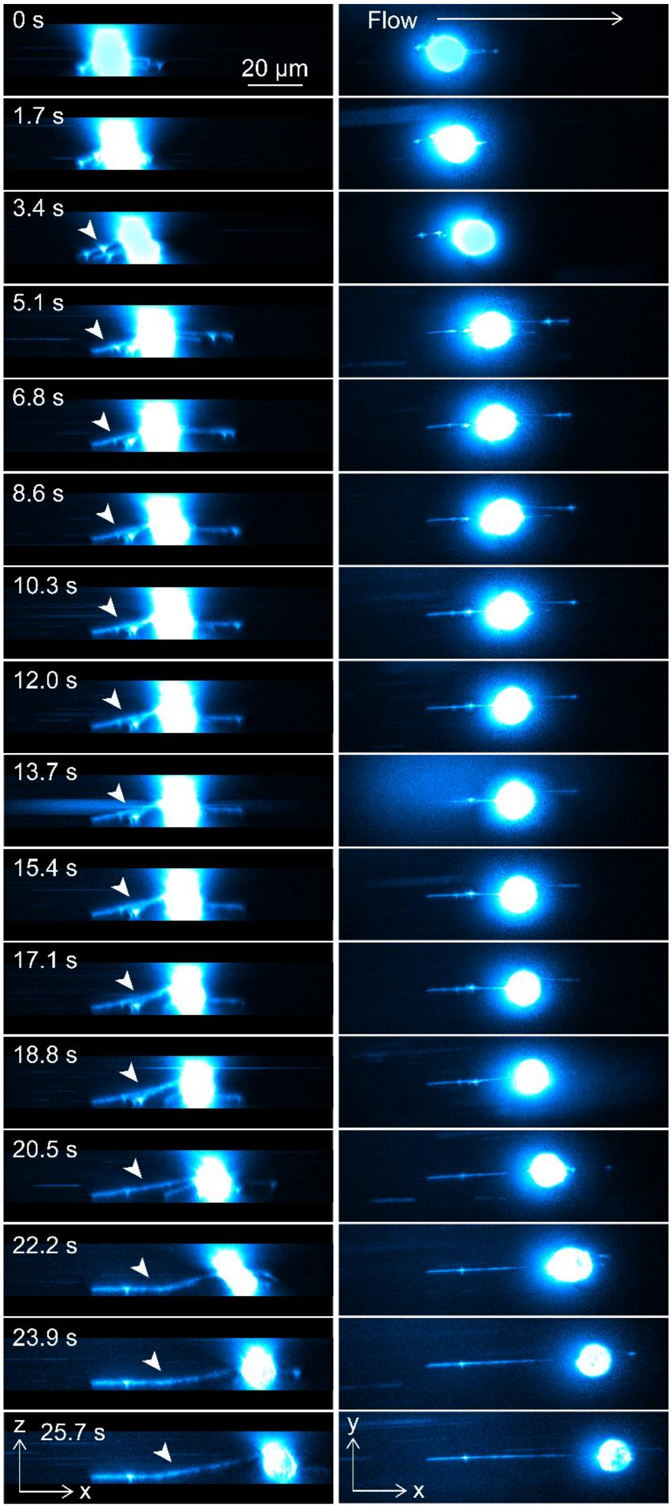


**Supplementary Figure 17. 3D views of the elongation of the tether formed on a KG1a cell rolling over E-selectin.** Side view (left) and top view (right) of the 3D reconstructed time-lapse

fluorescence images of CD44 (immunostained by Alexa-Fluor-488-conjugated anti-CD44 antibody, clone 515) captured during cell rolling over surface-deposited rh E-selectin molecules. The 3D images were reconstructed by recording fluorescence images of the cell at 57 different Z-axis positions with 0.5 μm step size. The arrowheads indicate the tether that shows elongation behavior. The cells were injected into the chambers at a shear stress of 2 dyne $cm^{-2}$ (0.2 Pa).

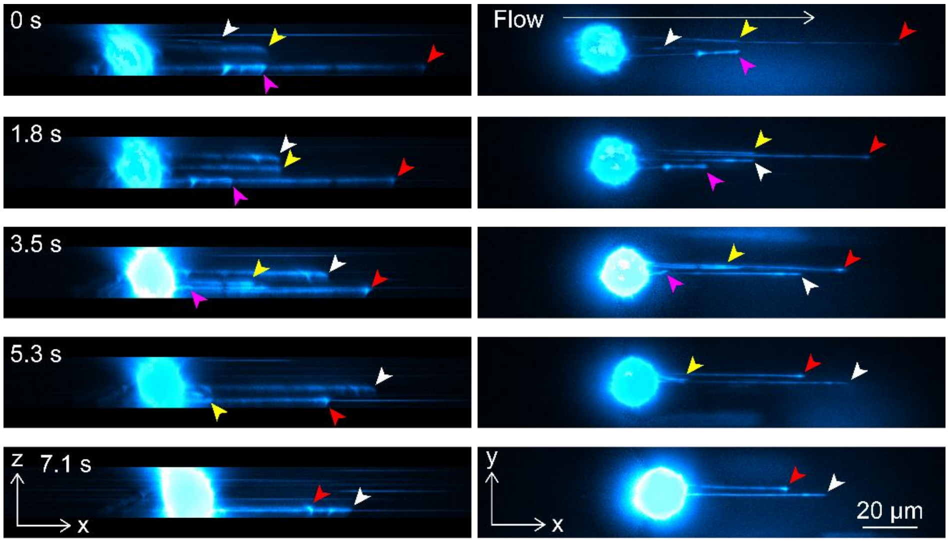


**Supplementary Figure 18. 3D views of the slings formed on a KG1a cell that show changes in the Z-axis positions during cell rolling over E-selectin.** Side view (left) and top view (right) of the 3D reconstructed time-lapse fluorescence images of CD44 (immunostained by Alexa-Fluor-488-conjugated anti-CD44 antibody, clone 515) captured during cell rolling over the surface-deposited rh E-selectin molecules. The 3D images were reconstructed by recording fluorescence images of the cell at 59 different Z-axis positions with 0.5 μm step size. The arrow heads in white, yellow, red and magenta show four different slings formed during cell rolling. The cells were injected into the chambers at a shear stress of 2 dyne $cm^{-2}$ (0.2 Pa).

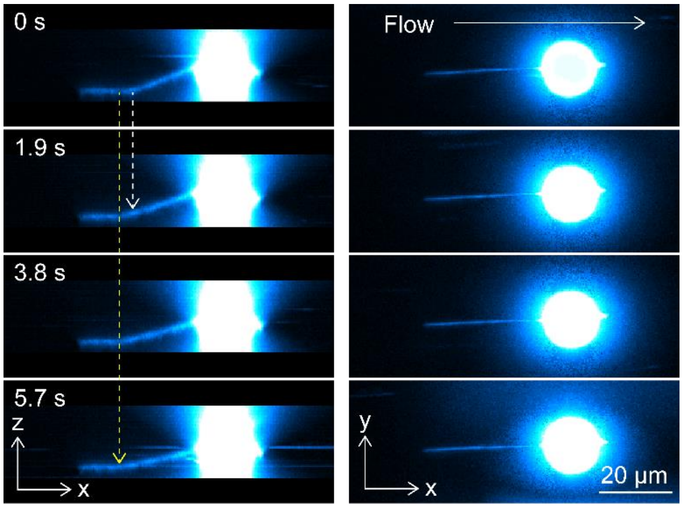


**Supplementary Figure 19. 3D views of the tether formed on a KG1a cell that show changes in the position of the anchoring points during cell rolling over E-selectin.** Side view (left) and top view (right) of the 3D reconstructed time-lapse fluorescence images of CD44 (immunostained by Alexa-Fluor-488-conjugated anti-CD44 antibody, clone 515) captured during cell rolling over the surface-deposited rh E-selectin molecules. The 3D images were reconstructed by recording fluorescence images of the cell at 63 different Z-axis positions with 0.5 μm step size. The white and yellow arrows show the positions of the two anchoring points formed on the tether. The first anchoring point (white arrow) is detached during cell rolling, whereas the second anchoring point (yellow arrow) continues attaching to the surface E-selectin. The cells were injected into the chambers at a shear stress of 2 dyne $cm^{-2}$ (0.2 Pa).

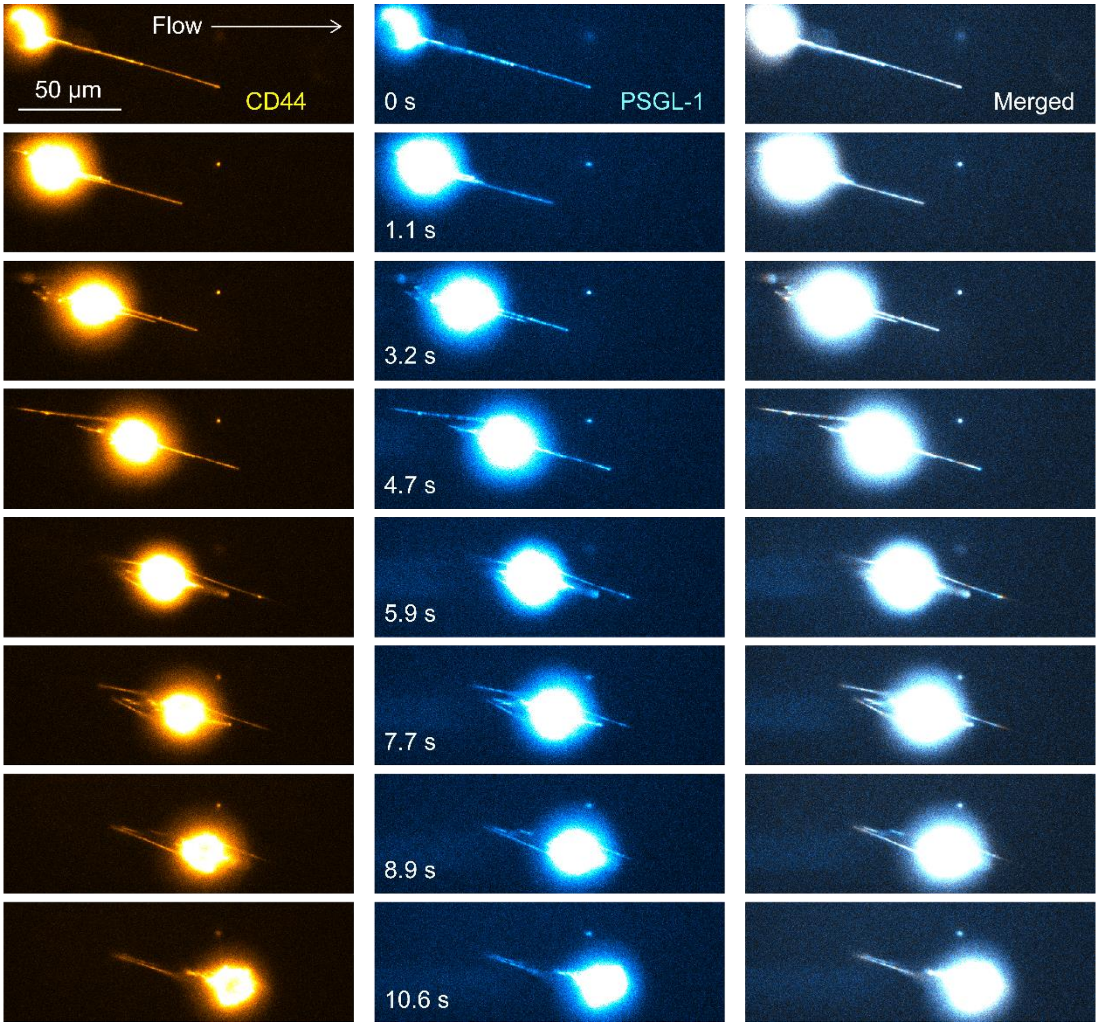


**Supplementary Figure 20. Colocalization of CD44 and PSGL-1 on the tethers and slings formed on a KG1a cell rolling over E-selectin.** Time-lapse fluorescence images of CD44 (yellow, immunostained by Alexa-Fluor-647-conjugated anti-CD44 antibody, clone 515) and PSGL-1 (cyan, immunostained by Alexa-Fluor-488-conjugated anti-PSGL-1 antibody, clone KPL-1) captured during cell rolling over the surface-deposited rh E-selectin molecules. The merged images are displayed in the right panels. The cells were injected into the chambers at a shear stress of 2 dyne cm$^{-2}$ (0.2 Pa).

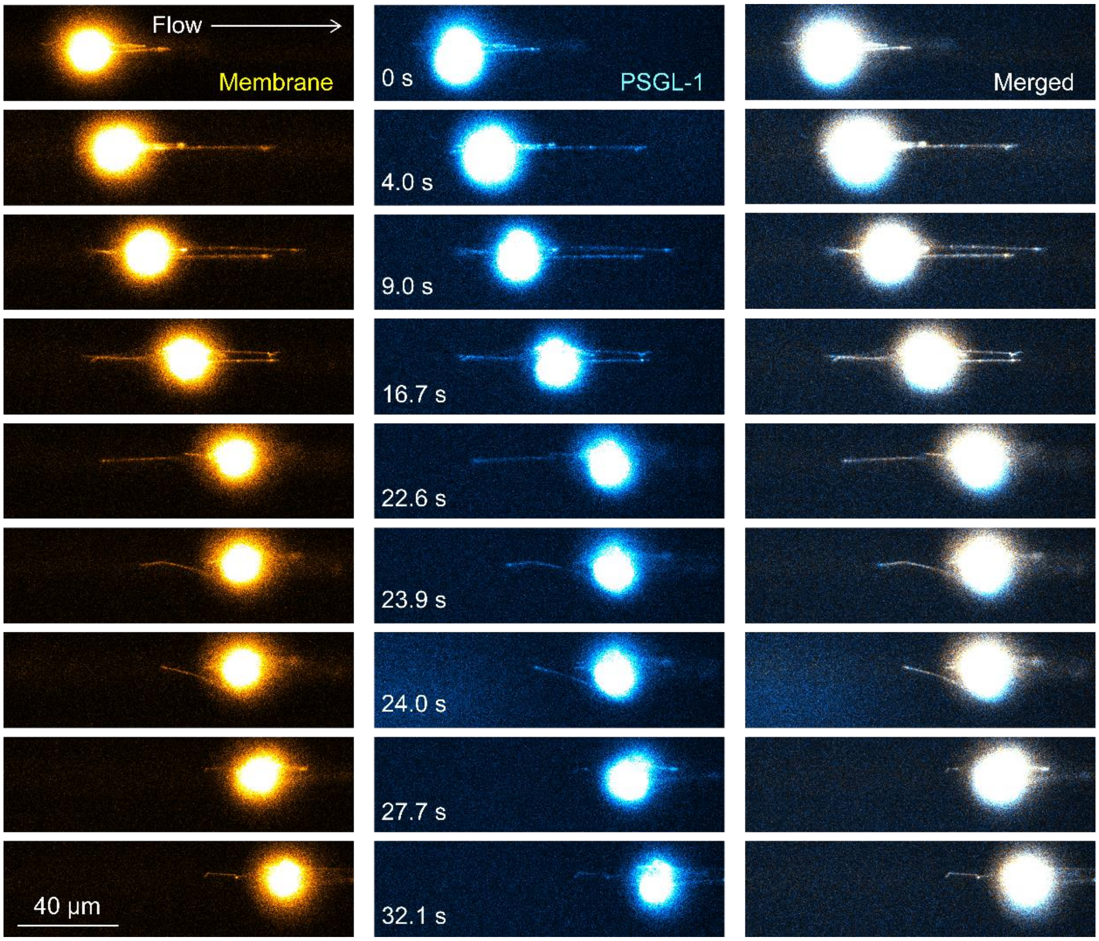


**Supplementary Figure 21. Colocalization of cell membrane and PSGL-1 on the tethers and slings formed on a KG1a cell rolling over E-selectin.** Time-lapse fluorescence images of the cell membrane (yellow, stained by Vybrant DiO dye) and PSGL-1 (cyan, immunostained by Alexa-Fluor-647-conjugated anti-PSGL-1 antibody, clone KPL-1) captured during cell rolling over the surface-deposited rh E-selectin molecules. The merged images are displayed in the right panels. The cells were injected into the chambers at a shear stress of 2 dyne cm$^{-2}$ (0.2 Pa).

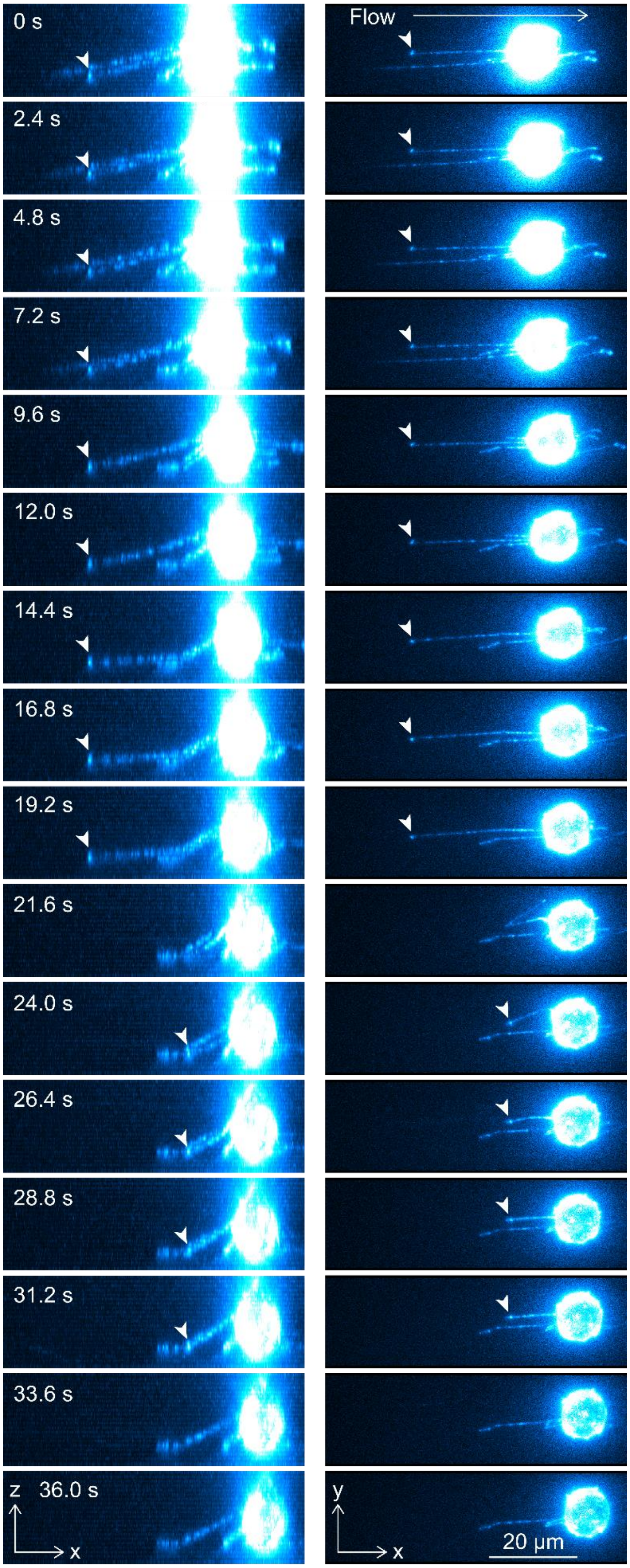
0 s
2.4 s
4.8 s
7.2 s
9.6 s
12.0 s
14.4 s
16.8 s
19.2 s
21.6 s
24.0 s
26.4 s
28.8 s
31.2 s
33.6 s
z 36.0 s
x
Flow
y
x
20 μm

**Supplementary Figure 22. 3D views of the tethers and slings formed on a KG1a cell that demonstrates clustering behavior of PSGL-1 at the tethering points during cell rolling over E-selectin.** Side view (left) and top view (right) of the 3D reconstructed time-lapse fluorescence images of PSGL-1 (immunostained by Alexa-Fluor-555-conjugated anti-PSGL-1 antibody, clone KPL-1) captured during cell rolling over the surface-deposited rh E-selectin molecules. The 3D images were reconstructed by recording fluorescence images of the cell at 40 different Z-axis positions with 1.0 μm step size. The arrowheads show the bright fluorescence spots of PSGL-1 (i.e. clusters of PSGL-1) found at the tethering points. The cells were injected into the chambers at a shear stress of 2 dyne $cm^{-2}$ (0.2 Pa).

(a)

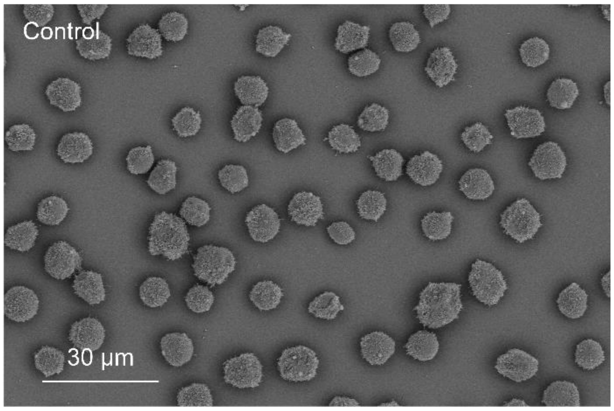


(b)

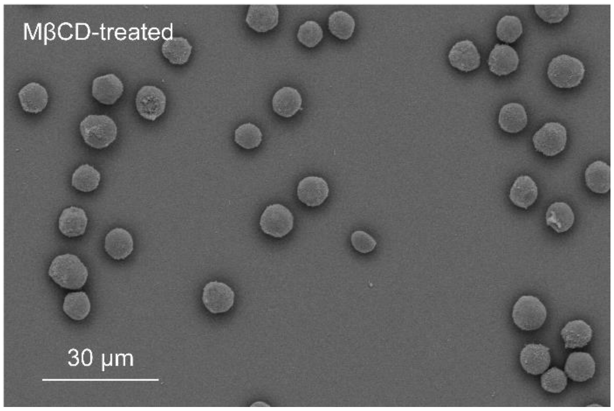


**Supplementary Figure 23. Scanning electron microscopy (SEM) images of KG1a cells.** SEM images of **(a)** control and **(b)** methyl-β-cyclodextrin (MβCD)-treated KG1a cells. These are representative images of n=2 independent experiments.

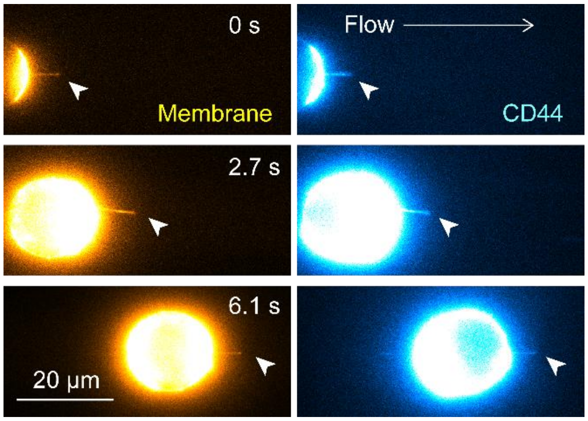


**Supplementary Figure 24. Spatial colocalization of CD44 and membrane tethers/slings on the MβCD-treated KG1a cells.** Two-color fluorescence images of the cell membrane (stained by Vybrant DiO dye) and CD44 (immunostained by Alexa-Fluor-647-conjugated anti-CD44 antibody clone 515) captured during the MβCD-treated KG1a cell rolling over the surface-deposited rh E-selectin molecules. White arrowheads show slings formed during the cell rolling. The cells were injected into the chambers at a shear stress of 2 dyne $cm^{-2}$ (0.2 Pa).

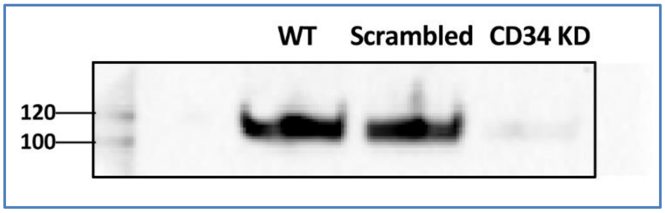


**Supplementary Figure 25. CD34 knockdown in KG1a cells.** KG1a cells were transfected with either a scrambled control siRNA (Silencer Select, Life technologies; 4390843) or with siRNA specific for CD34 (Select, Life technologies; 4392420-s2644). Cells were collected after 48 h of transfection and subjected to Western blot analysis for CD34 protein expression. Lane 1: untreated KG1a cells; Lane 2: scrambled negative control siRNA; Lane 3: CD34 KD KG1a cells. Membrane bands were normalized using total cells protein. This is representative of n=5 independent experiments.

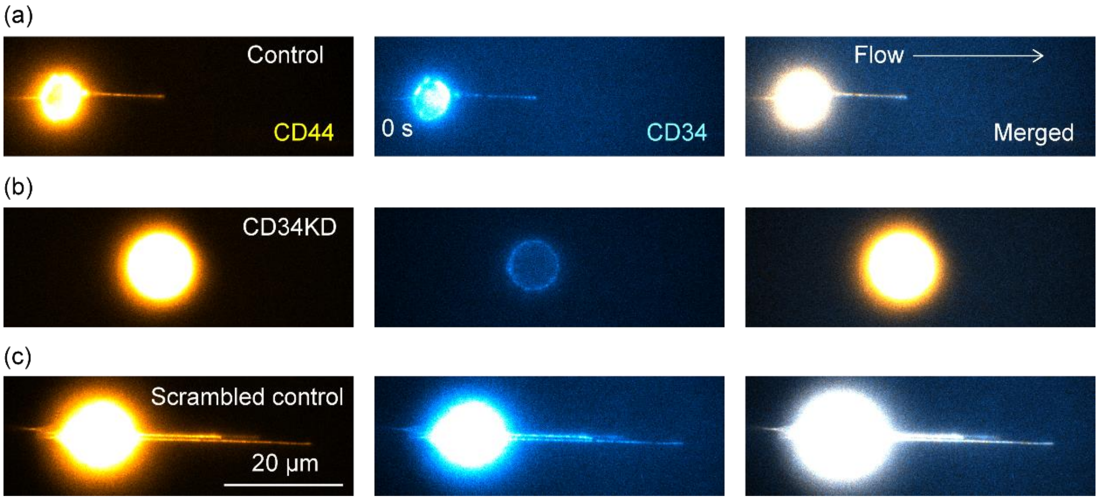


**Supplementary Figure 26. Effect of the knockdown of CD34 on the formation of tethers and slings on KG1a cells while rolling over E-selectin.** Examples of the two-color fluorescence images of CD34 (cyan, immunostained by Alexa-Fluor-488-conjugated anti-CD34 antibody clone QBEND/10) and CD44 (yellow, immunostained by Alexa-Fluor-647-conjugated anti-CD44 antibody clone 515) on **(a)** control KG1a cell, **(b)** KG1a cell transfected with siRNA specific for CD34, and **(c)** KG1a cell transfected with scrambled control siRNA captured during cell rolling over the surface-deposited rh E-selectin molecules. The cells were injected into the chambers at a shear stress of 2 dyne $cm^{-2}$ (0.2 Pa). These images were captured using identical imaging conditions and were shown in an identical image contrast.

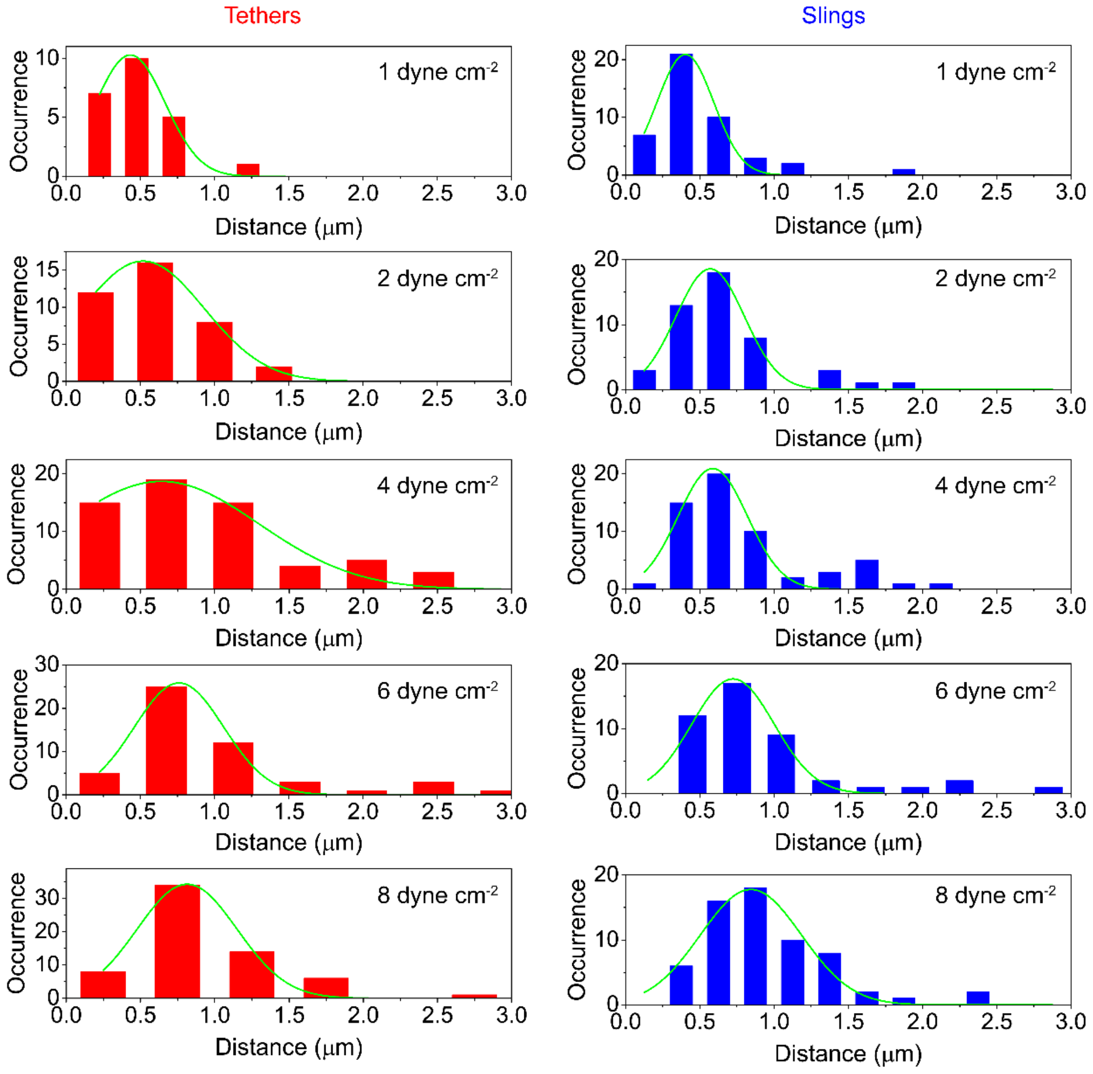


**Supplementary Figure 27. Distance between the PSGL-1 spots on the tethers and slings formed on KG1a cells rolling over E-selectin.** Frequency histograms of the distance between the adjacent fluorescent spots of PSGL-1 (immunostained by Alexa-Fluor-555-conjugated anti-PSGL-1 antibody, clone KPL-1) on the tethers (red bars) and slings (blue bars) formed during the KG1a cells rolling over rh E-selectin. The cells were injected into the chambers at a shear stress of either 1, 2, 4, or 8 dyne cm$^{-2}$ (0.1, 0.2, 0.4, or 0.8 Pa).

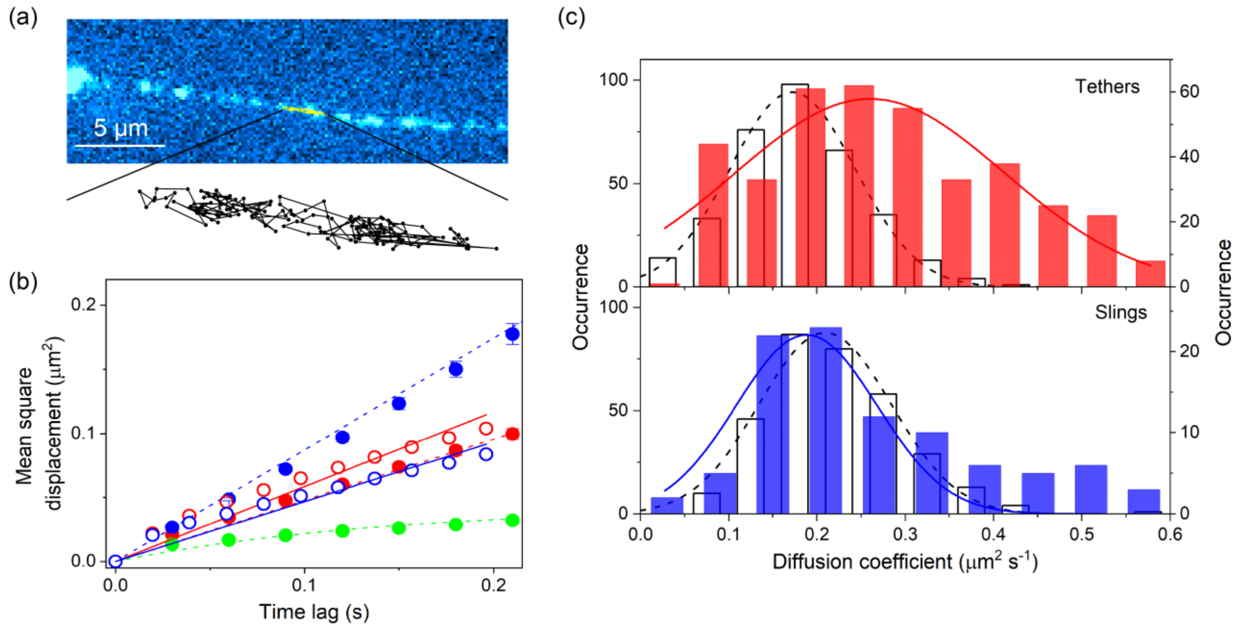


**Supplementary Figure 28. Single-molecule tracking analysis of PSGL-1 on the tethers and slings using anti-PSGL-1 Fab fragment.** (a) Single-molecule fluorescence images of PSGL-1 (immunostained by Atto532-conjugated anti-PSGL-1 Fab) on the sling of the KG1a cell formed during cell rolling over E-selectin at a shear stress of 2 dyne cm$^{-2}$ (0.2 Pa). An example of the single-molecule diffusion trajectories obtained from the time-lapse fluorescence images is shown by the yellow line. (b) Mean square displacement (MSD) versus time lag plots obtained for the PSGL-1 molecules diffusing on the tethers (red open circles) and slings (blue open circles) captured by using the Atto532-conjugated anti-PSGL-1 Fab. The MSD versus time lag plots obtained using the Alexa-Fluor-555-conjugated anti-PSGL-1 whole antibody are displayed as a reference (red circles, blue circles, and green circles for the PSGL-1 molecules on tethers, slings, and microvilli, respectively). (c) Frequency histograms of the diffusion coefficient of the PSGL-1 molecules on the tethers (top, red) and slings (bottom, blue) of the KG1a cells formed during cell rolling over E-selectin at a shear stress of 2 dyne cm$^{-2}$ (0.2 Pa). The diffusion coefficients were calculated by fitting the MSD plots obtained from the individual diffusion trajectories to Eq. 4.

The solid lines show Gaussian fittings. The diffusion data obtained using the the Alexa-Fluor-555-conjugated anti-PSGL-1 whole antibody are displayed as a reference (black bars and dashed lines for the frequency histograms and Gaussian fittings, respectively).

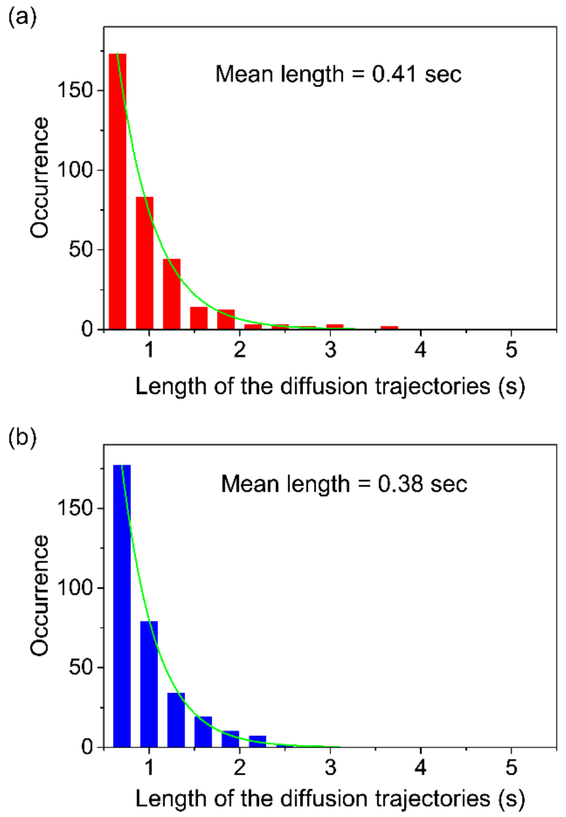


**Supplementary Figure 29. Length of the single-molecule diffusion trajectories of the PSGL-1 molecules on the tethers and slings formed on KG1a cells rolling over E-selectin.** Frequency histograms of the length of the single-molecule diffusion trajectories of PSGL-1 molecules (immunostained by Alexa-Fluor-555-conjugated anti-PSGL-1 antibody, clone KPL-1) that show diffusional motion on the **(a)** tethers and **(b)** slings formed during the KG1a cells rolling over rh E-selectin. The solid lines show the fitting to single-exponential decaying function. The mean trajectory lengths (0.41s and 0.38 s for the trajectories obtained from the tethers and slings, respectively) were calculated using the decay constants obtained by the fits. The cells were injected into the chambers at a shear stress of 2 dyne $cm^{-2}$ (0.2 Pa).

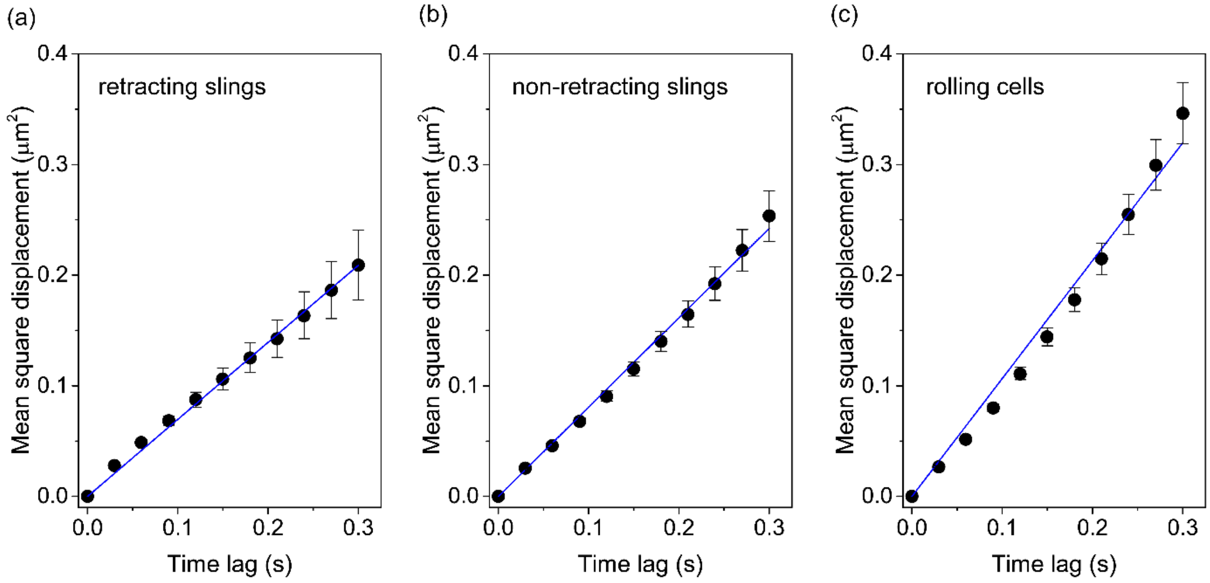


**Supplementary Figure 30. MSD analysis of the PSGL-1 molecule diffusing on the slings.** The MSD versus time lag plots were calculated using single-molecule diffusion trajectories of the PSGL-1 molecules diffusing on the **(a)** retracting slings without the effect of the cell rolling, **(b)** stable slings without the effect of the cell rolling and **(c)** stable slings with the effect of the cell rolling. The error bars show the standard errors of the mean determined by 47, 169 and 104 MSD plots obtained for the PSGL-1 molecules diffusing on the retracting slings, non-retracting slings and stable slings with the effect of the cell rolling, respectively.

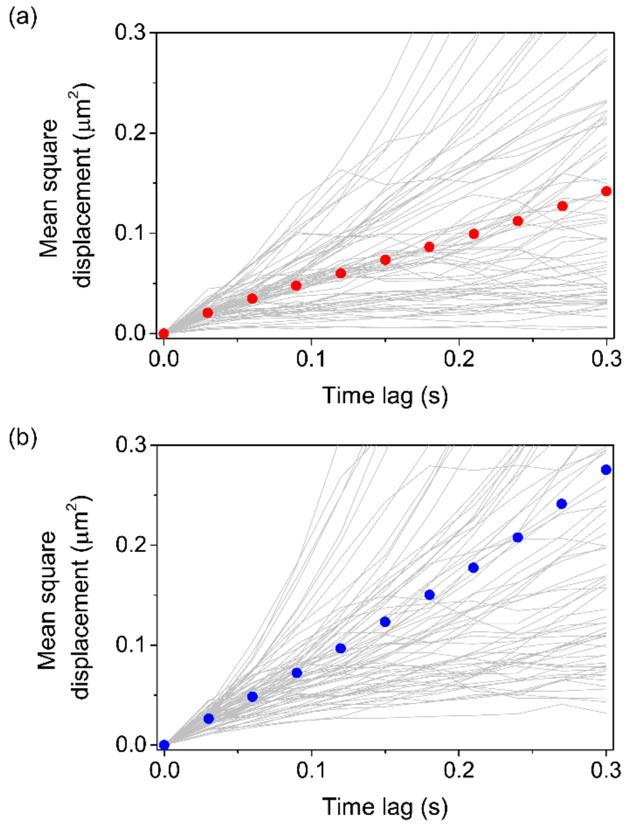


**Supplementary Figure 31. MSD vs time-lag plots obtained from the single-molecule diffusion trajectories of PSGL-1 on the tethers and slings.** MSD vs time-lag plots obtained for PSGL-1 molecules (immunostained by Alexa-Fluor-555-conjugated anti-PSGL-1 antibody, clone KPL-1) that show diffusional motion on the **(a)** tethers and **(b)** slings formed during KG1a cells rolling over rh E-selectin. The grey lines show individual MSD vs time-lag plots obtained from individual single-molecule diffusion trajectories. The red and blue dots show mean MSD vs time-lag plots for the tethers and slings, respectively. The cells were injected into the chambers at a shear stress of 2 dyne $cm^{-2}$ (0.2 Pa).

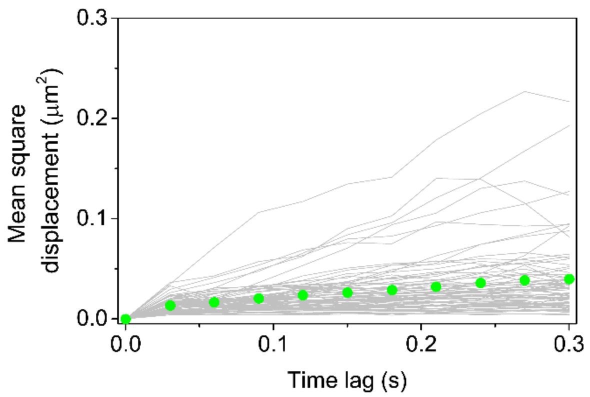


**Supplementary Figure 32. MSD vs time-lag plots obtained from the single-molecule diffusion trajectories of PSGL-1 localised on microvilli.** MSD vs time-lag plots obtained for PSGL-1 molecules (immunostained by Alexa-Fluor-488-conjugated anti-PSGL-1 antibody, clone KPL-1) that are localized on microvilli in the control KG1a cells. The grey lines show individual MSD vs time-lag plots obtained from individual single-molecule diffusion trajectories. The green dots show mean MSD vs time-lag plot. The cells were injected into uncoated microfluidic chambers without any recombinant E-selectin molecules. The fluorescence images were captured in the absence of any external shear stress.

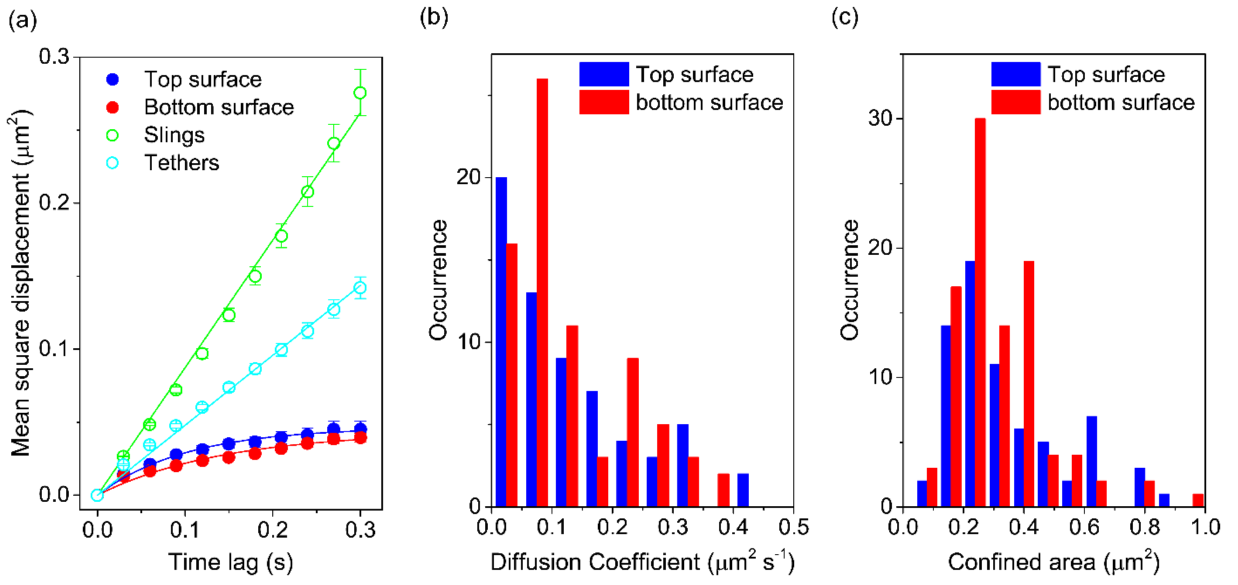


**Supplementary Figure 33. MSD analysis of the PSGL-1 molecule localised on the microvilli.** (a) MSD versus time lag plots calculated using single-molecule diffusion trajectories of the PSGL-1 molecules localized on the microvilli of the control KG1a cells at the top (blue) and bottom (red) surface of the cells. The error bars show the standard errors of the mean determined by 126 and 129 MSD plots obtained for the PSGL-1 molecules localized on the microvilli of the control KG1a cells at the top and bottom surface of the cells. The MSD versus time lag plots obtained from the single-molecule diffusion trajectories of the PSGL-1 molecules diffusing on the tethers (cyan) and slings (green) are also displayed as a comparison. Frequency histograms of the (b) diffusion coefficient and (c) confined area of the PSGL-1 molecules on the microvilli of the control KG1a cells at the top (blue) and bottom (red) surface of the cells.

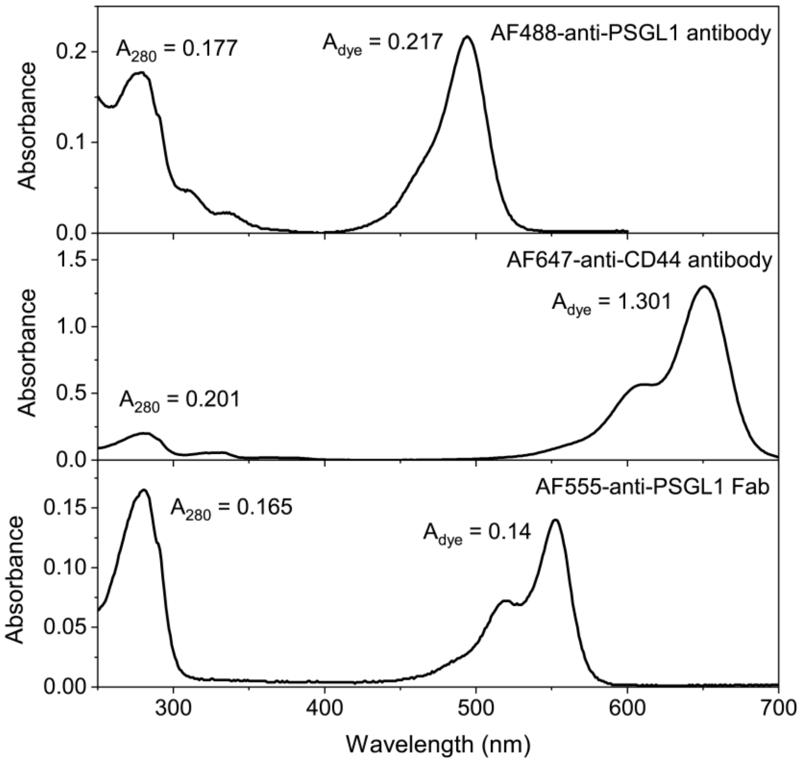


**Supplementary Figure 34. Absorption spectra of Alexa-Fluor-conjugated antibodies**. Alexa-Fluor-488-conjugated anti-PSGL-1 antibody clone KPL-1 (top), Alexa-Fluor-647-conjugated anti-CD44 antibody clone 515 (middle), and Alexa-Fluor-555-conjugated anti-PSGL-1 Fab fragment clone KPL-1 (bottom). $A_{280}$ and $A_{dye}$ denote the absorbance at 280 nm and the absorbance at the peak absorption of the dyes.

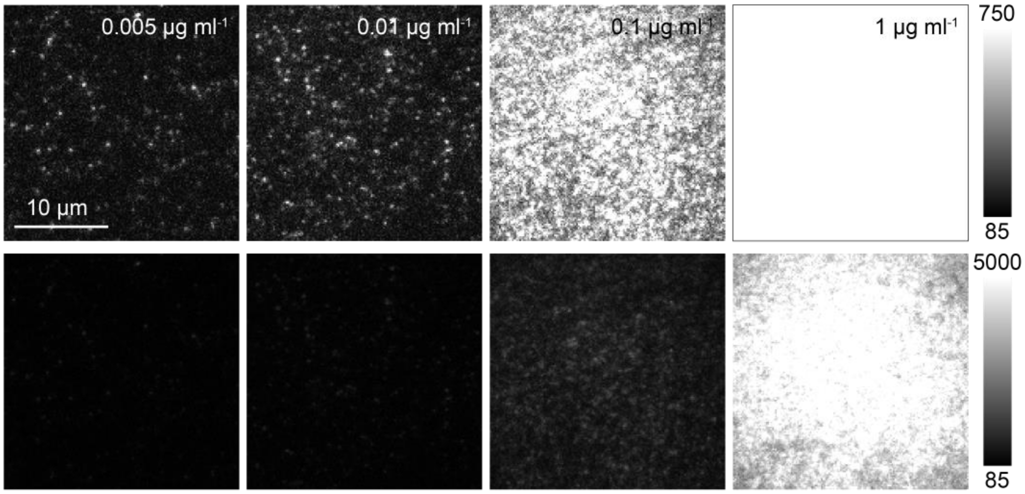


**Supplementary Figure 35. Fluorescence images of the Alexa-Fluor-647-conjugted anti-PSGL-1 antibody deposited on cover slips.** The concentrations of the anti-PSGL-1 antibody in the solutions for the deposition are indicated in each image. The top and bottom images are the same fluorescence images with different image contrasts, which are indicated in the color bars displayed in the right.

## Supplementary Notes

### Supplementary note 1. Bivalency effect of the antibodies

Antibodies have two binding sites to their epitopes. This may cause artificial clustering of the labeled molecules. Since we observed the discrete spatial distribution of the PSGL-1 molecule on the tethers and slings of the KG1a cell that are formed during the cell rolling over E-selectin, we investigated if this spatial distribution is a result of the artificial clustering of the immunostained PSGL-1 molecules due to the bivalency effect of the anti-PSGL-1 antibody. To that end, we immunostained PSGL-1 on KG1a cells using the Alexa-Fluor-555-conjugated Fab fragment of the anti-PSGL-1 antibody that has only one binding site to its epitope. The immunofluorescence image of PSGL-1 on the tethers and slings of the KG1a cells obtained using the Alexa-Fluor-555-conjugated Fab fragment of the anti-PSGL-1 antibody showed a spatial distribution of the PSGL-1 molecules on the tethers and slings very similar to that obtained using the Alexa-Fluor-555-conjugated anti-PSGL-1 antibody (i.e. whole antibody with two binding sites) (Supplementary Figure 6). This result demonstrates that the discrete spatial distribution of the PSGL-1 molecules on the tethers and slings are not the result of artificial clustering of PSGL-1 due to the bivalency effect of the antibody.

### Supplementary note 2. Effect of the immunostaining of PSGL-1and CD44 expressed on the KG1a cells on their binding specificity to E-selectin

To the best of our knowledge and through experiments done in our lab and others, antibodies against glycoprotein ligands, such as PSGL-1 and CD44, that can block binding to E-selectin have not been described or reported.[1, 2] Indeed real time immunoprecipitation studies performed in our lab have shown that these antibodies do not block the ability of the immunoprecipitated protein to

recognize E-selectin.[2] To further illustrate this point that the CD44 or PSGL-1 antibodies used in these studies are not blocking the binding of E-selectin to the E-selectin ligands, we performed a flow cytometry experiment where we either stained KG1a cells with recombinant E-selectin followed by an anti-human IgG Fc antibody conjugated to Alexa-Fluor-647 (Blue plot), or pre-treated the cells with an antibody to CD44 (515 clone; green) or PSGL-1 (KPL1 clone; red) followed by staining with recombinant E-selectin (Supplementary Figure 6). As is evident in Supplementary Figure 6 and much like previous data from our lab, we did not observe a decrease in the ability of the cells to bind E-selectin if the red and green plots are compared to the blue plot.

**Supplementary note 3. Density of CD44 on the tethers and slings**

We calculated the number of the CD44 molecules per unit length of slings in a way similar to that for PSGL-1 (see Methods). The calculated densities of CD44 on the tethers and slings are in the range of 24 – 111 molecules $\mu m^{-1}$ (i.e. the distances between adjacent CD44 molecules on the tethers and slings are in the range of 9 – 42 nm). This result demonstrates quantitatively that the densities of CD44 on the tethers and slings are much higher than those of PSGL-1 (0.4 – 0.8 µm distances between adjacent molecules on the tethers and slings, i.e. 1.25 – 2.5 molecules $\mu m^{-1}$). The distances between adjacent CD44 molecules on the tethers and slings estimated by the density calculation (9 – 42 nm) are much shorter than the spatial resolution of our fluorescence microscope (approximately 200 nm according to Rayleigh criterion). Therefore, individual CD44 molecules on the tethers and slings cannot be resolved in our fluorescence imaging experiment. This is consistent with the contiguous distribution of CD44 observed in our imaging experiment. The density of CD44 molecules on the tethers and slings decreased with an increase in the shear stress (Supplementary Figure 9). Similar shear stress-dependent density was observed for PSGL-1 (Figure 6g).

**Supplementary note: 4. Comparison of E-selectin and P-selectin in terms of the tether formation**

We observed the formation of the tethers with mean lengths of 18 – 30 μm at shear stresses ranging between 1 and 8 dyne $cm^{-2}$ (0.1 and 0.8 Pa) (Figure 3b and 6f). Although the formation of tethers has been reported for neutrophils rolling over P-selectin,[3] relatively short tethers (mean lengths of approximately 10 μm) were observed only at high shear stresses (above 6 dyne $cm^{-2}$ (0.6 Pa)).[4] Although we cannot rule out the possibility that cell type dependent elastic properties of cell membranes may affect the tethering behaviour, it is more likely that the difference is due to the strength of selectin-ligand interactions. Our observations suggest that E-selectin-ligand interactions are much stronger than P-selectin-ligand interactions. Since rapture force of E-selectin-ligand interactions observed for polymorphonuclear leukocytes is similar to that of P-selectin-ligand interactions,[5] it is likely that the difference in the binding strength between E-selectin and P-selectin is a result of different dissociation constants against their ligands. The stronger binding (i.e. smaller dissociation rate) of the tethers resists shear stresses for longer periods of time before they dissociate from the surface selectins, and thus causes the formation of longer tethers. This stronger binding is consistent with previous studies from our lab illustrating that the $k_{off}$ of binding between selectin ligands and E-selectin is low.[2, 6]

**Supplementary note 5. Elastic property of the tethers and slings and its contribution to cell rolling**

The very bright and contiguous fluorescence of CD44 from the tethers and slings (Figure 3c) allows detailed discussion about the dynamic behaviour of the tethers and slings. Our live-cell fluorescence imaging experiment clearly captured the flipping of the broken tethers from the rear side of the KG1a cell to its front side during rolling on E-selectin (Figure 3c). The images demonstrate that the flipping of the tethers occurred at a time scale of approximately 240 ms. This

time scale is longer than that reported for neutrophils rolling on P-selectin (less than 100 ms),[4] although similar tethers and slings were observed in both cell types. Given much larger shear stress (10 dyne $cm^{-2}$ (1 Pa)) used in the experiment on neutrophils compared with our experiment (Figure 3c, 6 dyne $cm^{-2}$ (0.6 Pa)), the observed difference is likely to be due to the different flow velocities in these experiments rather than different elastic properties of the tethers formed in these experiments. These results indicate that the elastic properties of the tethers and slings that make these structures so flexible are similar in different cell types, implying the existence of common mechanisms in the formation of tethers and slings.

**Supplementary note: 6. Colocalisation of CD44 and membrane tethers/slings on MβCD-treated KG1a cells**

The two-color fluorescence imaging experiment of the membrane stain and CD44 on the control KG1a cells showed perfect spatial colocalization of CD44 and the cell membrane in the tethers and slings (Figure 2a), and thus the spatiotemporal behavior of the tethers and slings (e.g. length of the tethers and slings at different applied shear stresses) can be characterized by analyzing fluorescence images obtained for CD44. The MβCD treatment of the cells may alter the spatial localization of CD44, which may affect the analysis of the dynamic behavior of the tethers and slings. We evaluated this by capturing two-color fluorescence images of the membrane stain and CD44 on the MβCD-treated KG1a cells Supplementary Figure 24). While the tethers and slings of the MβCD-treated cells formed during cell rolling over E-selectin were much shorter than those of the control cells (Figure 5e), we found perfect spatial colocalization of the membrane stain and CD44 (Supplementary Figure 24). This result confirms that the spatiotemporal behavior of the tethers and slings on the MβCD-treated cells can be characterized by analyzing fluorescence images obtained for CD44.

**Supplementary note 7. Effect of the immunostaining of PSGL-1 on its single-molecule tracking analysis**

In order to evaluate the effect of the immunostaining of PSGL-1 on its single-molecule tracking analysis, we conducted single-molecule tracking experiments of the PSGL-1 molecules on the tethers and slings using Fab fragments conjugated to Atto532 dyes (Supplementary Figure 28a). The mean square displacement vs. time lag plots obtained for the PSGL-1 molecules on the tethers and slings were similar to those obtained using the whole antibodies (Supplementary Figure 28b). Furthermore, the frequency histograms of the diffusion coefficient obtained by the mean square displacement analysis agree well with the data obtained using the whole antibodies (Supplementary Figure 28c). We note that the frequency histograms obtained using the Fab fragments are slightly broader compared with those obtained using the whole antibodies. This is mainly due to the less bright fluorescence obtained using the Fab fragments that resulted in less accurate localization and tracking of the molecules. Overall, our results suggest that the single-molecule tracking analysis could be conducted using the whole antibodies without introducing associated artifacts.

**Supplementary note 8. Effect of the retraction of the slings and rolling of the cells on the single-molecule diffusion analysis of PSGL-1**

The single-molecule tracking analysis of the PSGL-1 molecules on the slings could be affected by the motion of the slings (i.e. either stable or retracting) and the cell (i.e. either rolling within the acquisition time of the diffusion trajectory or not). Thus, we split the diffusion trajectories into three categories and calculated the mean square displacement (MSD) versus time lag plots separately.

A. PSGL-1 molecules diffusing on the stable slings without the effect of the cell rolling.

B. PSGL-1 molecules diffusing on the retracting slings without the effect of the cell rolling.

C. PSGL-1 molecules diffusing on the stable slings with the effect of the cell rolling.

While we observed slight differences between the three cases, in principle, all the MSD versus time lag plots showed linear relationship (Supplementary Figure 30). This result confirms the random-mode diffusion of the PSGL-1 molecules on the slings. The effect of the retraction of the slings and the cell rolling is negligible in our MSD analysis probably because of the decoupling of the time scale of the diffusional motion of the PSGL-1 molecules and the motion of the slings and the cells.

**Supplementary note 9. Effect of the three dimensional diffusion along the tethers and slings on the single-molecule tracking analysis**

We captured the diffusion of single PSGL-1 molecules at 30 Hz at a single focal plane (i.e., 2D imaging). Since the PSGL-1 molecules diffuse along tethers and slings, the diffusion does not always occur in the focal plane of the microscope. In order to avoid the defocusing issue (due to the 3D diffusion), we captured the images of tethers and slings that were placed parallel to the surface (Supplementary Movie 4 and 5). This was easy for the slings since many slings were stretched by a laminar flow and aligned parallel to the surface (Supplementary Movie 5). On the other hand, as the reviewer pointed, it was not possible to capture the diffusion of the single molecules along the tethers that do not have any anchoring points (i.e., tethers attached to the surface only at the tethering point). Thus, we obtained the single molecule diffusion data on the tethers by capturing the images of the tethers that attached to the surface at multiple points (i.e., tethering point and anchoring points, Supplementary Movie 4). This allowed us to visualize the entire diffusional motion without the effect of defocusing. In addition, the means square

displacement (MSD) plots obtained for the PSGL-1 molecules diffusing on the tethers and slings strongly suggest the random diffusion, not a confined diffusion (i.e., linear relationship between time lag and MSD, Figure 7b blue and red plots). If the single-molecule diffusion analysis is affected by the defocusing issue, we should observe a confined motion. Thus, our data rather suggests that the analysis was not affected by defocusing issue. In contrast, the PSGL-1 molecule localized on microvilli of the control cells showed a confined diffusion (Figure 7b green plot). The PSGL-1 molecules localized on microvilli stay at the focus during the entire data acquisition time (as the length of microvilli is less than 1 micrometer), and therefore this data suggest that the diffusion of PSGL-1 on microvilli is indeed confined motion.

**Supplementary note 10. Effect of the surface on the single-molecule tracking analysis and the interpretation of the obtained data**

The single-molecule imaging experiment of the PSGL-1 molecules that are localized on the microvilli of the control KG1a cells (i.e. not rolling cells) was conducted by placing the immunostained cells on the surface of the microfluidic chamber. Although we did not deposit any molecules that have a specific interaction with the PSGL-1 molecules (e.g. E-selectin), the PSGL-1 molecule may interact with the surface in a nonspecific manner, and therefore the diffusional motion of the PSGL-1 may be affected. We investigated this effect by analysing the single-molecule diffusion trajectories obtained from both the bottom and top surfaces of the cells. To minimize the effect of the refractive index mismatch between the cell samples and the immersion media, we used the silicone immersion objective lens (60× NA = 1.3, UPLSAPO60XS2) for this imaging experiment. The MSD versus time lag plots obtained from these experiments showed very similar behavior, including smaller diffusion coefficient compared with that obtained from the PSGL-1 molecules on the tethers and slings and confined-mode diffusion (Supplementary Note

Figure 5a). Quantitative analysis of the MSD versus time lag plots revealed that the PSGL-1 molecules localized on the microvilli at both the bottom and top surface of the cells show similar diffusion coefficient and the confined size (Supplementary Note Figure 5b, 5c). These results confirm that there is a negligible effect of the surface on the diffusional motion of the PSGL-1 molecules localised on microvilli. We used the data obtained from the bottom surface of the cells for the analysis as we obtained slightly better quality of the single-molecule fluorescence images of the PSGL-1 molecules from the bottom surface of the cells.

The diffusion coefficient of the PSGL-1 molecules localized on the microvilli of the control KG1a cells obtained from the MSD analysis (0.1 $\mu m^2 s^{-1}$) is approximately 30 fold larger than the diffusion coefficient of PSGL-1 reported previously using fluorescence recovery after photobleaching (FRAP) technique (0.003 $\mu m^2 s^{-1}$).[7] Given the different time and length scales of the diffusional motion captured by these two methods, it is very likely that we captured the motion of microvilli rather than the diffusional motion of the PSGL-1 molecules in our single-molecule fluorescence imaging experiment (i.e. the PSGL-1 molecules stay at the tip of microvilli during the image acquisition and therefore we capture the motion of the microvilli through the fluorescence signal of the PSGL-1 molecules). This is strongly supported by the fact that the size of the confinement area (0.29 $\mu m^2$ that corresponds to the confinement length of approximately 0.54 μm) estimated by the MSD analysis is close to the size of the area expected to be covered by the microvilli since our SEM experiments on the KG1a cells revealed that the length of the microvilli is in the range of several hundred of nanometers (Figure 5c). Also, the length of the microvilli on neutrophils has been estimated to be about 0.3 μm.[8] Therefore, the real diffusion coefficient of the PSGL-1 molecules localised on the microvilli would be much smaller than our estimation by the MSD analysis of the single-molecule diffusion trajectories and may be closer to

the value determined by the FRAP technique. The difference in the diffusion coefficient obtained in our experiment and in the previous study may also be partly explained by differences from cell to cell.

**Supplementary note 11. Density of labeling and fluorescence brightness of the antibodies**

We experimentally determined the average number of fluorophores conjugated to the antibodies (i.e., density of labeling (DOL)). DOL was calculated using the following equations,

$$C = \frac{[A_{280} - (A_{\text{dye}} - CF_{280})]}{\varepsilon_{\text{AB}}} \tag{S1}$$

$$DOL = \frac{A_{\text{dye}}}{\varepsilon_{\text{dye}} \times C} \tag{S2}$$

where $C$ and $\varepsilon_{\text{AB}}$ are the concentration and the molar extinction coefficient of the antibody at 280 nm, respectively. $A_{280}$ and $A_{\text{dye}}$ denote the absorbance of the dye-antibody conjugate at 280 nm and at the peak absorption wavelength for the respective dye, respectively. $\varepsilon_{\text{dye}}$ and $CF_{280}$ are the molar extinction coefficient of the conjugated dye and a correction factor for the fluorophore's contribution to the absorbance at 280 nm. According to the manufacturer of the dyes used in this study, $\varepsilon_{\text{dye}}$ and $CF_{280}$ are $\varepsilon_{\text{dye}}$ = 71,000 and $CF_{280}$ = 0.11 for Alexa Fluor 488, $\varepsilon_{\text{dye}}$ = 150,000 and $CF_{280}$ = 0.08 for Alexa Fluor 555, and $\varepsilon_{\text{dye}}$ = 239,000 and $CF_{280}$ = 0.03 for Alexa Fluor 647. $\varepsilon_{\text{AB}}$ of the whole antibody and the Fab fragment are 203000 and 75000 $M^{-1}$ $cm^{-1}$, respectively. Therefore, the DOL for AF488-anti-PSGL-1 and AF647-anti-CD44 antibodies displayed in Supplementary Figure 34 top and middle are estimated to be DOL = 4.1 and 6.8, respectively. DOL for AF555-anti-PSGL-1 antibody (Fab fragment) is estimated to be DOL = 0.46 (Supplementary Figure 34 bottom). We experimentally determined DOL in each conjugation reaction because DOL in each reaction was not constant. DOL for the whole antibodies was in the range of DOL = 4-9.

After the conjugation reaction, we experimentally determined the fluorescence brightness obtained from single dye-conjugated anti-PSGL-1 and anti-CD44 antibodies (using the dye-conjugated antibodies mentioned above, Supplementary Figure 35). The dye-conjugated anti-PSGL-1 antibody deposited on a glass surface at the high concentration (1 μg $ml^{-1}$ in Supplementary Figure 34) showed bright and uniform fluorescence. On the other hand, at lower concentrations (in particular at 0.005 and 0.01 μg $ml^{-1}$ concentration in Figure 30), spatially isolated diffraction-limited fluorescence spots were observed. This concentration dependence strongly suggested that each fluorescence spot appeared in these images is a single dye-conjugated anti-PSGL-1 antibody. We experimentally determined the average fluorescence brightness of the single dye-conjugated anti-PSGL-1 antibody using these images. We determined the average fluorescence brightness of the single dye-conjugated anti-CD44 antibody in a similar way. We note that the average fluorescence brightness of the single dye-conjugated anti-PSGL-1 and anti-CD44 antibodies was experimentally determined for each batch of the dye-conjugated antibodies because DOL in each reaction was not constant. We also note that the presence of multiple fluorophores (on average 4-9 dyes per antibody) resulted in brighter fluorescence signal from the immunostained cells, which allowed us to quantitatively characterize the spatiotemporal dynamics of the selectin ligands (e.g., estimation of the number of the molecules in each spot along the tethers and slings).

## Supplementary References


1. Merzaban, J. S.; Burdick, M. M.; Gadhoum, S. Z.; Dagia, N. M.; Chu, J. T.; Fuhlbrigge, R. C.; Sackstein, R., Analysis of glycoprotein E-selectin ligands on human and mouse marrow cells enriched for hematopoietic stem/progenitor cells. *Blood* **2011,** *118* (7), 1774-1783.

2. AbuSamra, D. B.; Al-Kilani, A.; Hamdan, S. M.; Sakashita, K.; Gadhoum, S. Z.; Merzaban, J. S., Quantitative characterization of E-selectin interaction with native CD44 and P-selectin glycoprotein ligand-1 (PSGL-1) using a real time immunoprecipitation-based binding assay. *J. Biol. Chem.* **2015,** *290* (35), 21213-21230.

3. Sundd, P.; Gutierrez, E.; Koltsova, E. K.; Kuwano, Y.; Fukuda, S.; Pospieszalska, M. K.; Groisman, A.; Ley, K., 'Slings' enable neutrophil rolling at high shear. *Nature* **2012,** *488* (7411), 399-403.

4. Marki, A.; Gutierrez, E.; Mikulski, Z.; Groisman, A.; Ley, K., Microfluidics-based side view flow chamber reveals tether-to-sling transition in rolling neutrophils. *Sci Rep* **2016,** *6*, 28870.

5. Hanley, W. D.; Wirtz, D.; Konstantopoulos, K., Distinct kinetic and mechanical properties govern selectin-leukocyte interactions. *J. Cell Sci.* **2004,** *117* (12), 2503-2511.

6. AbuSamra, D. B.; Aleisa, F. A.; Al-Amoodi, A. S.; Jalal Ahmed, H. M.; Chin, C. J.; Abuelela, A. F.; Bergam, P.; Sougrat, R.; Merzaban, J. S., Not just a marker: CD34 on human hematopoietic stem/progenitor cells dominates vascular selectin binding along with CD44. *Blood Adv* **2017,** *1* (27), 2799-2816.

7. Gaborski, T. R.; Clark, A.; Waugh, R. E.; McGrath, J. L., Membrane Mobility of beta 2 Integrins and Rolling Associated Adhesion Molecules in Resting Neutrophils. *Biophys. J.* **2008,** *95* (10), 4934-4947.

8. Shao, J. Y.; Ting-Beall, H. P.; Hochmuth, R. M., Static and dynamic lengths of neutrophil microvilli. *Proc. Natl. Acad. Sci. U. S. A.* **1998,** *95* (12), 6797-6802.
