# Supplementary material for: Single-molecule imaging and microfluidic platform reveal molecular mechanisms of leukemic cell rolling
Source: Commun Biol. 2021 Jul 14;4:868. doi: 10.1038/s42003-021-02398-2 (PMC8280113; doi:10.1038/s42003-021-02398-2)
Supplement: Supplementary file 3 — Descriptions of Additional Supplementary Files [file 42003_2021_2398_MOESM3_ESM.pdf]

Description of Additional Supplementary Files

File name: Supplementary Movie 1
Description: Time-lapse fluorescence images of CD44 on a KG1a cell perfused into the rh E-selectin-deposited microfluidic chamber at a shear stress of 1 – 8 dyne cm-2 (1 – 8 Pa). The CD44 molecules were immunostained by Alexa-Fluor-647-conjugated anti-CD44 antibody, clone 515. Scale bar = 20 µm.

File name: Supplementary Movie 2
Description: Time-lapse fluorescence images of CD44 on a KG1a cell perfused into the rh E-selectin-deposited microfluidic chamber that shows the conversion of a tether into sling. The CD44 molecules were immunostained by Alexa-Fluor-647-conjugated anti-CD44 antibody, clone 515. The cells were injected into the chambers at a shear stress of 6 dyne cm-2 (0.6 Pa). Scale bar = 20 µm.

File name: Supplementary Movie 3
Description: Time-lapse fluorescence images of CD44 on a KG1a cell perfused into the rh E-selectin-deposited microfluidic chamber that shows the retraction of the sling. The CD44 molecules were immunostained by Alexa-Fluor-647-conjugated anti-CD44 antibody, clone 515. The cells were injected into the chambers at a shear stress of 8 dyne cm-2 (0.8 Pa). Scale bar = 20 µm.

File name: Supplementary Movie 4
Description: Time-lapse fluorescence images of PSGL-1 on a KG1a cell perfused into the rh E-selectin-deposited microfluidic chamber that shows the discrete spatial distribution of the PSGL-1 molecules on the tether. The PSGL- molecules were immunostained by AlexaFluor-555-conjugated anti-PSGL-1 antibody, clone KPL-1. The cells were injected into the chambers at the shear stress of 2 dyne cm-2 (0.2 Pa). Scale bar = 10 µm.

File name: Supplementary Movie 5
Description: Time-lapse fluorescence images of PSGL-1 on a KG1a cell perfused into the rh E-selectin-deposited microfluidic chamber that shows the discrete spatial distribution of the PSGL-1 molecules on the sling. The PSGL- molecules were immunostained by Alexa-Fluor555-conjugated anti-PSGL-1 antibody, clone KPL-1. The cells were injected into the chambers at a shear stress of 2 dyne cm-2 (0.2 Pa). Scale bar = 10 µm.

File name: Supplementary Movie 6
Description: Time-lapse fluorescence images of CD44 on KG1a cells perfused into the rh E-selectin-deposited microfluidic chamber that shows the formation of the tethers and slings on all the rolling cells. The CD44 molecules were immunostained by Alexa-Fluor-647-conjugated anti-CD44 antibody, clone 515. The cells were injected into the chambers at a shear stress of 4 dyne cm-2 (0.4 Pa). Scale bar = 20 µm.

File name: Supplementary Movie 7
Description: Time-lapse fluorescence images of PSGL-1 on a KG1a cell perfused into the rh E-selectin-deposited microfluidic chamber that shows the merger of multiple tethers. The PSGL-molecules were immunostained by Alexa-Fluor-555-conjugated anti-PSGL-1 antibody, clone KPL-1. The cells were injected into the chambers at a shear stress of 2 dyne cm-2 (0.2 Pa). Scale bar = 5 µm.

File name: Supplementary Data 1

Description: Source data for Figure 3b, Figure 4e, Figure 5g, Figure 6e, 6f, 6g, Figure 7b, Figure 7c,
Figure 7e, and Figure 7f.
