# Supplementary material for: Single-molecule imaging and microfluidic platform reveal molecular mechanisms of leukemic cell rolling
Source: Commun Biol. 2021 Jul 14;4:868. doi: 10.1038/s42003-021-02398-2 (PMC8280113; doi:10.1038/s42003-021-02398-2)
Supplement: Supplementary file 12 — Reporting Summary [file 42003_2021_2398_MOESM12_ESM.pdf]

nature research

Corresponding author(s): Satoshi Habuchi and Jasmeen S. Merzaban

Last updated by author(s): Jun 14, 2021

Reporting Summary

Nature Research wishes to improve the reproducibility of the work that we publish. This form provides structure for consistency and transparency in reporting. For further information on Nature Research policies, see Authors & Referees and the Editorial Policy Checklist .

Statistics

For all statistical analyses, confirm that the following items are present in the figure legend, table legend, main text, or Methods section.

n/a	Confirmed	
☐	☒	The exact sample size (*n*) for each experimental group/condition, given as a discrete number and unit of measurement
☐	☒	A statement on whether measurements were taken from distinct samples or whether the same sample was measured repeatedly
☐	☒	The statistical test(s) used AND whether they are one- or two-sided<br>*Only common tests should be described solely by name; describe more complex techniques in the Methods section.*
☒	☐	A description of all covariates tested
☒	☐	A description of any assumptions or corrections, such as tests of normality and adjustment for multiple comparisons
☐	☒	A full description of the statistical parameters including central tendency (e.g. means) or other basic estimates (e.g. regression coefficient) AND variation (e.g. standard deviation) or associated estimates of uncertainty (e.g. confidence intervals)
☐	☒	For null hypothesis testing, the test statistic (e.g. *F*, *t*, *r*) with confidence intervals, effect sizes, degrees of freedom and *P* value noted<br>*Give P values as exact values whenever suitable.*
☒	☐	For Bayesian analysis, information on the choice of priors and Markov chain Monte Carlo settings
☒	☐	For hierarchical and complex designs, identification of the appropriate level for tests and full reporting of outcomes
☒	☐	Estimates of effect sizes (e.g. Cohen's *d*, Pearson's *r*), indicating how they were calculated

Our web collection on statistics for biologists contains articles on many of the points above.

Software and code

Policy information about availability of computer code

Data collection	Fluorescence images of the cells were recorded using Andor iQ3 software. Bright-field images of the cells were recorded using CellSens software. SEM images of the cells were recorded using XT Server Microscope. Flow cytometry data was recorded using FlowJo7.6.1 software.
Data analysis	Fluorescence images were analyzed using Image J software. Super-resolution fluorescenece images were reconstructed using Localizer software. Rolling behavior of the cells was analyzed using TrackMate, an Image J plugin. MSD plots of PSGL-1 molecules on the cells were obtained using a custom written Matlab program.

For manuscripts utilizing custom algorithms or software that are central to the research but not yet described in published literature, software must be made available to editors/reviewers. We strongly encourage code deposition in a community repository (e.g. GitHub). See the Nature Research guidelines for submitting code & software for further information.

Data

Policy information about availability of data

All manuscripts must include a data availability statement. This statement should provide the following information, where applicable:

- Accession codes, unique identifiers, or web links for publicly available datasets
- A list of figures that have associated raw data
- A description of any restrictions on data availability

The authors declare that [the/all other] data supporting the findings of this study are available within the paper [and its supplementary information files].

Field-specific reporting

Please select the one below that is the best fit for your research. If you are not sure, read the appropriate sections before making your selection.

[x] Life sciences   [ ] Behavioural & social sciences   [ ] Ecological, evolutionary & environmental sciences

For a reference copy of the document with all sections, see nature.com/documents/nr-reporting-summary-flat.pdf

Life sciences study design

All studies must disclose on these points even when the disclosure is negative.

Sample size	No statistical methods were used to predetermine the sample sizes.
Data exclusions	No data were excluded.
Replication	All experiments were repeated at least twice to assure reproducibility. All the single cell fluorescence microscopy images reported in this study are representative examples of multiple (n > 2) independent experiments . Further, key behaviors of the cell that were reported in this study (i.e., length of tethers and slings, number of PSGL-1 and CD44 molecules on tethers and slings, diffusion coefficients and modes of PSGL-1 molecules) were statistically verified.
Randomization	Randomization was not used in this study.
Blinding	Blinding was not done in this study.

Reporting for specific materials, systems and methods

We require information from authors about some types of materials, experimental systems and methods used in many studies. Here, indicate whether each material, system or method listed is relevant to your study. If you are not sure if a list item applies to your research, read the appropriate section before selecting a response.

Materials & experimental systems

n/a	Involved in the study	
[ ]	[x]	Antibodies
[ ]	[x]	Eukaryotic cell lines
[x]	[ ]	Palaeontology
[x]	[ ]	Animals and other organisms
[x]	[ ]	Human research participants
[x]	[ ]	Clinical data

Methods

n/a	Involved in the study	
[x]	[ ]	ChIP-seq
[ ]	[x]	Flow cytometry
[x]	[ ]	MRI-based neuroimaging

Antibodies

Antibodies used	Provided in the Method ssections
Validation	All antibodies are commercially available and have been verified by the manufacturers. Further, CD34 antibody was validated by loss of band on Western blot analysis of CD34 knockdown cells with siRNA. CD44 and PSGL-1 antibodies were validated by isotype control in flow cytometric analysis and well as in single-cell fluorescence imaging analysis.

Eukaryotic cell lines

Policy information about cell lines

Cell line source(s)	KG1a cell line was purchased from ATCC. Primary human CD34posHSPCs isolated from umbilical cord blood and mononuclear cell from whole cord blood were purchased from ALL Cells.
Authentication	Authentication was performed by the vendors.
Mycoplasma contamination	Cell lines were certified as mycoplasma negative from the vendor (ATCC); cell lines were maintained and routinely tested for mycoplasma using MycoFluor Mycoplasma Detection Kit (M-7006; Molecular Probes).
Commonly misidentified lines (See ICLAC register)	None.

Flow Cytometry

Plots

Confirm that:

- [x] The axis labels state the marker and fluorochrome used (e.g. CD4-FITC).
- [x] The axis scales are clearly visible. Include numbers along axes only for bottom left plot of group (a 'group' is an analysis of identical markers).
- [x] All plots are contour plots with outliers or pseudocolor plots.
- [x] A numerical value for number of cells or percentage (with statistics) is provided.

Methodology

Sample preparation	Provided in the Method sections
Instrument	FACSCanto flow cytometer (Beckman Dickenson)
Software	FlowJo7.6.1
Cell population abundance	CD34 positive hematopoietic stem/progenitor primary cells were sorted from cord blood mononuclear cells (CB-MNC) as described in the methods section. The cell percentage was around 30% of whole CB-MNCs and the purity was 98% after sorting. 200,000 KG1a cells and CD34 positive HSPCs were stained and at least 10,000 cell events were recoded by the FACSCanto flow cytometer.
Gating strategy	The stained cells with specific conjugated antibody were normalized to the isotype control antibody that stain the same cell type under same buffer conditions. Positive events were determined after reading the cells with isotype control.

- [x] Tick this box to confirm that a figure exemplifying the gating strategy is provided in the Supplementary Information.
